# Benchmarking bacterial taxonomic classification using nanopore metagenomics data of several mock communities

**DOI:** 10.1038/s41597-024-03672-8

**Published:** 2024-08-10

**Authors:** Alexander Van Uffelen, Andrés Posadas, Nancy H. C. Roosens, Kathleen Marchal, Sigrid C. J. De Keersmaecker, Kevin Vanneste

**Affiliations:** 1 Transversal activities in Applied Genomics, Sciensano, Brussels, Belgium; 2 Department of Information Technology, Internet Technology and Data Science Lab (IDLab), Interuniversity Microelectronics Centre (IMEC), Ghent University, Ghent, Belgium; 3 Department of Plant Biotechnology and Bioinformatics, Ghent University, Ghent, Belgium; 4 Department of Genetics, University of Pretoria, Pretoria, South Africa

**Keywords:** Classification and taxonomy, Metagenomics

## Abstract

Taxonomic classification is crucial in identifying organisms within diverse microbial communities when using metagenomics shotgun sequencing. While second-generation Illumina sequencing still dominates, third-generation nanopore sequencing promises improved classification through longer reads. However, extensive benchmarking studies on nanopore data are lacking. We systematically evaluated performance of bacterial taxonomic classification for metagenomics nanopore sequencing data for several commonly used classifiers, using standardized reference sequence databases, on the largest collection of publicly available data for defined mock communities thus far (nine samples), representing different research domains and application scopes. Our results categorize classifiers into three categories: low precision/high recall; medium precision/medium recall, and high precision/medium recall. Most fall into the first group, although precision can be improved without excessively penalizing recall with suitable abundance filtering. No definitive ‘best’ classifier emerges, and classifier selection depends on application scope and practical requirements. Although few classifiers designed for long reads exist, they generally exhibit better performance. Our comprehensive benchmarking provides concrete recommendations, supported by publicly available code for reassessment and fine-tuning by other scientists.

## Introduction

Metagenomics considers the study of genetic material from uncultured microorganisms by sequencing^1^. Through directly sequencing the DNA in a sample, metagenomics allows detecting a wide range of microorganisms and their corresponding genes without isolation, cultivation or any *a priori* knowledge. Metagenomics therefore has the potential to be assumption-free and unbiased, rendering it possible to fully characterize the microbiome of a complex sample^2,3^. Moreover, without the need for isolation, it becomes possible to analyze organisms that are not cultivable or have specific (unknown) growth conditions. Consequently, metagenomics has been successfully employed in a wide array of domains in life sciences such as the study of the human gut^4^, sewage water^5^, soil and water quality^6^, rapid identification of the etiological agent, known or novel, in clinical settings^7^, and the detection of foodborne pathogens during outbreak investigation^8^.

All DNA is sequenced in shotgun metagenomics, whereas amplicon sequencing selectively targets marker genes within specific DNA regions, such as the 16S rRNA genes for bacteria and archaea, and the internal transcribed spacer regions (ITS) for fungi^2^. Significant improvements in sequencing technologies in recent years have made shotgun metagenomics a more attractive method for broader applications^9^. The most commonly used sequencing technology currently is Illumina, a second-generation sequencing technology. While Illumina provides massive parallel sequencing and a low error rate, it can typically only produce reads of lengths 100–300 bp. These short reads restrict investigating complex genomes and unraveling repetitive elements^10^. By contrast, long-read third-generation sequencing offers much longer reads spanning several thousand of bases and provides the possibility to characterize samples more accurately. An especially interesting platform is offered by Oxford Nanopore Technologies (ONT), through the release of their MinION device with a low cost per million reads, fast real-time sequencing and long read lengths up to several 10,000 s of bases^11^. Despite exhibiting higher error rates, significant improvements in recent years have reduced this error rate, and the new Q20 chemistry promises a modal 99% accuracy with reads length of around 10–30kb^12,13^. Pacific Biosciences (PacBio) constitutes an alternative long-read sequencing technology. While PacBio can offer higher accuracy through the generation of ‘high-fidelity’ reads, this advantage comes at the cost of read length^14^. Additionally, its higher capital investment and run costs may be a limiting factor^15^. Consequently, ONT has been increasingly adopted by the scientific community^16^, including for shotgun metagenomics applications^17–20^.

An essential step in many shotgun metagenomics applications is taxonomic classification, which assigns sequencing reads to specific taxonomic categories to identify the taxonomic groups they originate from. This is typically done by comparing sequencing reads to a database with reference sequences^21^. Both taxonomic classifiers and profilers are commonly used. A classifier assigns a taxonomic identification to each read by comparing it to a reference database filled with representative sequences. Examples of popular taxonomic classifiers used in this study include Kraken2^22^ and KMA^23^. In contrast, taxonomic profilers do not classify all reads but instead generate a taxonomic profile with estimates of taxonomic relative abundances, often based on clade-specific markers representing the distinctive signatures of species^21,24^. Examples of popular taxonomic profilers used in this study include MetaPhlAn3^25^ and mOTUs2^26^. Classifiers can be divided into DNA-to-DNA or DNA-to-protein methods depending on whether the reference database is composed of nucleic acid or protein sequences, respectively. DNA-to-protein methods are considered more sensitive towards new and highly variable sequences compared to DNA-to-DNA methods due to the degeneracy of the genetic code^27,28^. However, only reads with coding regions can be classified, and the complexity cost is higher as six reading frames need to be analyzed^28^. A profiler generates a taxonomic profile by relying on smaller taxon-specific regions and comprises a third category of DNA-to-marker methods that compare reads to a reference database containing clade-specific markers. Because these markers only occur in certain regions of the genome, a large fraction of reads will not be classified. The use of markers renders profilers less complex, although markers need to be continuously updated as newly sequenced genomes and species are reported to find representative clade-specific markers in new genomes^21^, and the potentially lower representativeness of DNA-to-marker databases hence could incur a performance cost^28^. The distinction between classifiers and profilers is not always clear-cut, as profilers typically employ read classification approaches to compute profiles, and the output of taxonomic classifiers can also be converted into profiles containing relative abundances^28^. Therefore, for the sake of convenience, we will refer to both methods as classifiers in this study. The rapidly increasing popularity of long-read sequencing has however not been on par with the development of new algorithms and applications specifically adapted for classification of long-read sequencing data. Consequently, taxonomic classification of long-read metagenomic data is often performed by tools designed for short reads, although there has been a recent increase in the number of long-read classification tools such as MetaMaps^29^, MEGAN-LR^30^ and deSAMBA^31^.

The choice of reference database to be used with the classifier is paramount. The detection of a certain species depends not only on the classifier’s ability, but also on the presence and the quality of a reference sequence for that species in the database. The classifier’s performance is hence dependent on the used database that should be as comprehensive as possible. However, increasing the number of reference sequences (and therefore completeness) can risk introducing sequences of lower quality, thus also increasing the chance for faulty taxonomic classifications due to more potential matches. A balance should therefore be struck between the completeness and quality of a reference database^28^, and using different databases can introduce unwanted biases when comparing classifiers^32–34^. Most classifiers include pre-built reference databases that are constructed from various sources. Comparing classifier performance using these default databases may yield differences not solely attributable to the classifier itself, but also to underlying reference database^34,35^. To eliminate database biases in benchmarking studies, a reference database should therefore ideally be used for each type of classifier that contains exactly the same sequences, although this is often impossible for DNA-to-marker methods because their databases are algorithmically constructed and specifically tailored to their associated classifiers.

With the increasing popularity of shotgun metagenomics, researchers rightfully saw the need for meaningful comparison and benchmarking to guide selecting the best taxonomic classifier to answer their research questions^28,36–42^. For instance, the Initiative for the Critical Assessment of Metagenome Interpretation (CAMI) is a community-driven effort that evaluates methods for metagenome analysis^43,44^ to establish standards for benchmark datasets, evaluation procedures and performance metrics. To evaluate performance, often synthetic, i.e., simulated, datasets are used. However, because metagenomic data is highly complex, synthetic datasets are likely to provide a simplified version, potentially missing key characteristics of the studied data^45^. Empirical data derived from defined mock communities (DMCs) therefore constitute a better alternative. A DMC is a well-defined and intentionally constructed mixture of known organisms. Sequencing DMCs has the advantage of knowing exactly what is expected, i.e., the ‘ground truth’ to which the output of the classifiers can be compared, and producing ‘real’ metagenomic data without potential biases from simulation^35,46^.

Efforts such as CAMI have provided valuable insights into classifier performance, but the majority of benchmarks have been focused on short-read data. Meyer *et al*.^43^ and McIntyre *et al*.^40^ included long-read data, but this evaluation was limited. Only few studies have evaluated taxonomic classification performance using long reads. Marić *et al*. evaluated thirteen tools on long-read data and concluded that the majority of tools, for both short and long reads, are prone to reporting organisms not present in the dataset^47^. However, only one ONT dataset originated from a real DMC (i.e., not simulated), and evaluation of performance was limited to a select few metrics without providing a broader systematic investigation. Portik *et al*. evaluated 11 tools and concluded that short-read classifiers required heavy filtering to achieve acceptable precision^24^. They found that long-read datasets produced better results than short-read datasets, but also included only one real DMC, sequenced with two different ONT chemistries. Additionally, they did not use the same database for the same type of classifier. Other benchmarking studies primarily focused on the nuances of the sequencing technology over the evaluation of classifiers, limiting both the quantity and depth of classifier assessments^48–51^. Consequently, little guidance is available for researchers wishing to perform taxonomic classification using long-read nanopore sequencing data.

In this paper, we extend the aforementioned studies through a systematic investigation of the performance of taxonomic classification of ONT data. We consider classifiers that fulfil three requirements: open-source, locally installable and allowing customization of the database. The underlying databases from the same type of classifier were harmonized to reduce biases introduced by using different reference databases. Performance was then evaluated in depth per classifier per sample, using a total of nine DMCs with different compositions, to provide a systematic evaluation of performance of nanopore long-read taxonomic classification.

## Methods

### Defined mock communities

An extensive literature search was conducted to find DMCs of microorganisms for which ONT data was publicly available, resulting in nine datasets, summarized in Table 1. For all nine DMCs, ONT data generated with the R9 technology was available, and for one DMC also data generated with the R10 technology. The samples ranged from containing a relatively limited number to a large number of species with different distributions. A sample with an ‘even’ distribution contained all species with equal relative abundances. A ‘staggered’ distribution indicated that the relative abundances differed, although equal abundances could occur for some species. Lastly, one sample had a logarithmic distribution for which each consecutive relative abundance was one-tenth of the previous one. Note that some DMCs were originally available as cells, whereas others were available as DNA. This information, together with in-depth detailed information per DMC on the exact species and their relative abundances, is available in Supplementary Table S3. Additionally, read length and mean read quality distribution plots are also available in the Supplementary Figures S8–S17. Since the DMCs were collected from different studies, they did not contain the same overall sequencing yield per sample. To ensure that differences in coverage per sample did not introduce any unwanted bias, each DMC was randomly subsampled to have the same number of total bases as the DMC with the lowest number of bases. Rasusa v0.7.1 was used to randomly downsample the DMCs to 3,125,920,499 bases with a seed set to 1^52^. Prior to downsampling, the R10 dataset was subsampled to match both the read length distribution and read count of the corresponding R9 dataset to remove the effect of read length on classification when comparing the R9 and R10 chemistry. Table 2 provides an overview of various sequence metrics before and after subsampling of the datasets. After downsampling, the reads of each DMC were filtered to only retain reads with a length higher than 1000 and a mean Phred score higher than 7.

**Table 1:** Used DMCs to benchmark the classifiers.

Name	Source	Catalog	Chemistry version	Composition	Distribution	Link	Run accession	DMC identifier
Zymo D6300	Zymo Research	D6300	R9	8 bacteria; 2 fungi	Even	10.1093/gigascience/giz043^71^	ERR2906227^72^	Zymo_D6300
Zymo D6310	Zymo Research	D6310	R9	8 bacteria; 2 fungi	Log	10.1093/gigascience/giz043^71^	ERR2906229^73^	Zymo_D6310
Zymo D6322	Zymo Research	D6322	R9	7 bacteria 1 fungus	Even, except the fungus	10.1038/s41592-022-01539-7^74^	ERR7255742^75^	Zymo_D6322
Zymo D6322	Zymo Research	D6322	R10	7 bacteria 1 fungus	Even, except the fungus	10.1038/s41592-022-01539-7^74^	ERR7287988^76^	Zymo_D6322_r10
Zymo D6331	Zymo Research	D6331	R9	1 archaea; 14 bacteria; 2 fungi	Staggered	10.1186/s40168-022-01415-8^17^	SRR17913200^77^	Zymo_D6331
Bei Resources HM-276D	Bei Resources	HM-276D	R9	20 bacteria	Even	10.1016/j.isci.2020.101223^78^	SRR11700265^79^	BeiRes_276D
Bei Resources HM-277D	Bei Resources	HM-277D	R9	20 bacteria	Staggered	10.1016/j.isci.2020.101223^78^	SRR11700264^80^	BeiRes_277D
Strain madness 1	Meslier, V., Quinquis, B., Da Silva, K. *et al*.	/	R9	22 archaea; 45 bacteria	Staggered	10.1038/s41597-022-01762-z^81^	ERR9765780^82^	StrainMad_1
Strain madness 2			R9	22 archaea; 61 bacteria			ERR9765781^83^	StrainMad_2
Strain madness 3			R9	14 archaea; 46 bacteria			ERR9765782^84^	StrainMad_3

The first column contains the dataset names. The second and third columns give the origin and catalog number (if applicable). The fourth column shows the nanopore chemistry used for sequencing. The fifth and sixth columns provide information on the composition of each dataset. The seventh column refers to the DOI number from where the datasets originate. The eighth column refers to the run accession number of the sequencing data. The last column is used a reference key to the DMCs throughout the text and figures. More detailed information on the exact composition of the DMCs, including their relative abundances, is available in Supplementary Table S3.

Abbreviations: DMC: Defined Mock Community; DOI: Digital Object Identifier.

**Table 2:** Sequence statistics of the DMCs.

	DMC identifier	Num seqs	Sum len	Min len	Avg len	Max len	Q1	Q2	Q3	N50	Q20 (%)	Q30 (%)
Raw	Zymo_D6300	3,491,078	14,007,156,825	5	4012.3	107972	1907	3275	5102	5213	15.18	0
	Zymo_D6310	3,667,007	16,032,264,247	5	4372	117224	2505	3822	5576	5290	13.34	0
	Zymo_D6322	8,851,918	31,995,546,765	15	3614.5	172125	561	1063	2782	13837	65.03	30.84
	Zymo_D6322_r10	18,831,686	53,221,766,826	10	2826.2	593108	490	1058	2663	7495	77.86	62.15
	Zymo_D6331	5,757,345	28,443,158,238	1000	4940.3	50325	3035	4572	6424	5913	65.26	25.44
	BeiRes_276D	11,610,183	35,578,375,166	5	3064.4	472762	660	1374	3244	6828	17.43	0
	BeiRes_277D	18,254,839	72,312,638,112	1	3961.3	214792	898	2065	4201	7857	39.24	10.06
	StrainMad_1	696,944	3,125,920,499	499	4485.2	45135	2072	4206	6311	6086	21.63	0
	StrainMad_2	831,802	3,690,876,744	499	4437.2	60869	2013	4154	6268	6057	21.63	0
	StrainMad_3	791,715	3,412,736,796	499	4310.6	42254	2070	4049	6033	5788	21.36	0
Downsampled	Zymo_D6300	779,595	3,125,921,589	5	4009.7	57563	1905	3276	5102	5212	15.16	0
	Zymo_D6310	714,975	3,125,926,503	5	4372.1	58449	2501	3823	5574	5292	13.35	0
	Zymo_D6322	864,519	3,125,936,623	84	3615.8	135502	561	1061	2779	13867	65.06	30.86
	Zymo_D6322_r10^*^	864,025	3,125,958,626	79	3617.9	157706	562	1063	2783	13927	78.23	62.66
	Zymo_D6331	632,692	3,125,922,651	1000	4940.7	34278	3032	4573	6422	5911	65.27	25.44
	BeiRes_276D	1,020,565	3,125,922,929	5	3062.9	219166	661	1376	3240	6817	17.41	0
	BeiRes_277D	789,968	3,125,921,897	1	3957	214792	897	2069	4200	7827	39.27	10.1
	StrainMad_1	696,944	3,125,920,499	499	4485.2	45135	2072	4206	6311	6086	21.63	0
	StrainMad_2	704,142	3,125,924,133	499	4439.3	60869	2012	4154	6273	6063	21.63	0
	StrainMad_3	725,300	3,125,922,602	499	4309.8	42254	2070	4048	6033	5787	21.36	0
	Zymo_D6300	779,595	3,125,921,589	5	4009.7	57563	1905	3276	5102	5212	15.16	0

The initial rows detail the raw DMCs, whereas the subsequent rows delineate the DMCs with subsampled reads, aiming for a total close to 3,125,920,499 bases. The second column shows the total number of reads, and the third column depicts the total number of bases. The fourth, fifth, and sixth columns display the minimum, average, and maximum sequence length, respectively. The seventh, eighth, and ninth columns represent the first quartile, median, and third quartile of read lengths. The tenth column denotes the N50 of the sequence length, and the eleventh and twelfth columns indicate the percentage of bases with quality scores greater than 20 and 30, respectively.

*Zymo_D6322_r10 was downsampled to match the read length distribution and read count of Zymo_D6322.

### Taxonomic classifiers

A literature review was performed to find classifiers commonly employed for or specifically designed for long-read data, encompassing also short-read methods frequently applied to long-read data. Every classifier considered for benchmarking is listed in Table 3. We imposed three rules on taxonomic classifiers included in our study. First, the classifier should be open-source. This ensures that the algorithm used is not a ‘black box’, can be peer-reviewed by others, and is free to increase accessibility. Second, it had to be possible to locally install the classifier to efficiently incorporate into in-house pipelines and safeguard ownership of any analyzed data. A local installation is also independent of external tools and remains unaffected by one’s internet speed or potential downtime. Third, the classifier should allow building a custom database compatible with the classifier to guarantee uniformity amongst reference databases and allow using reference sequences that may not be suitable for public sharing.

**Table 3:** Classifiers considered in this study.

Method type	Database	Classifier	Included in benchmark	Reason for exclusion
Short-read	DNA	Bracken^55^	Yes	—
	DNA	Centrifuge^56^	Yes	—
	DNA	Kraken2^22^	Yes	—
	Protein	Kaiju^27^	Yes	—
	Protein	MMseqs2^57^	Yes	—
	Marker	MetaPhlAn3^25^	Yes	—
	Marker	mOTUs2^26^	Yes	—
Long-read	DNA	BugSeq^53^.	No	Open-source, locally installable and custom database
	DNA	deSAMBA^31^	No	Custom database
	Protein	DIAMOND + MEGAN^54^	No	Custom database
	DNA	MetaMaps^29^	No	Custom database
Short and long-read	DNA	CCMetagen^58^	Yes	—
	DNA	KMA^23^	Yes	—

The first column shows the type of input the classifiers were designed to handle. The second column displays the employed reference database. The third column lists the classifiers. The fourth and fifth column indicate whether the classifier was included in this study, and why not if omitted.

Four classifiers specifically intended for long-read data were not included because they did not meet our inclusion criteria. Although deSAMBA^31^ has an option to build a custom database, the build time exceeded 60 days after which the process was manually killed. MetaMaps^29^ exhibited unsolvable errors during the database building that could not be resolved. BuqSeq^53^ was not evaluated because it violates all three rules. DIAMOND + MEGAN (community edition)^54^ requires to ‘meganize’ the output of DIAMOND before passing to MEGAN. This process needs a file that matches the alignments to the corresponding taxonomy, which can only be custom made when using the ultimate edition, which violates the custom database and open-source criteria.

Finally, we evaluated the performance of nine taxonomic classifiers. Six classifiers were originally created for short reads but also often used in studies for long reads: Bracken^55^, Centrifuge^56^, Kaiju^27^, Kraken2^22^, MetaPhlAn3^25^, and mOTUs2^26^. One classifier for long reads was included: MMSeqs2^57^. It should however be noted that MMSeqs2 was not specifically designed for long reads but rather for ‘metagenomic contigs’. Two classifiers were designed specifically for both short and long reads: KMA^23^ and CCMetagen^58^. All classifiers were used with their respective default parameters.

### Employed reference databases

For both the DNA-to-DNA and DNA-to-protein methods, a harmonized database approach was adapted, guaranteeing uniformity of databases among classifiers utilizing the same method. For DNA-to-DNA methods, the NCBI Reference Sequence Database (RefSeq)^59^, the most popular genomic and highly-curated reference database, served as the preferred genomic reference database. Following several curation steps, the employed genomic database contained 2,389,358 sequences, corresponding to 44,494 genomes and 20,219 unique species (see Supplementary for curation details). An overview of the content of this database is available Table 4. The following DNA-to-DNA classifiers were evaluated: Kraken2, Bracken, Centrifuge, KMA and CCMetagen (see Supplementary for details).

**Table 4:** Genomic reference sequences used for DNA-to-DNA methods.

Branch	Assembly level	Genomes	# Unique
Archaea	Complete Chromosome Scaffold	828	30 orders; 48 families; 160 genera; 563 species;
Bacteria	Complete	31559	193 orders; 474 families; 1754 genera; 8103 species;
Fungi	Complete Chromosome Scaffold	376	54 orders; 112 families; 192 genera; 369 species;
Protozoa	Complete Chromosome Scaffold	87	23 orders; 27 families; 36 genera; 86 species;
Viruses	Complete	11642	63 orders; 222 families; 1972 genera; 11096 species;
Other eukaryota (including human)	/	2	2 orders; 2 families; 2 genera; 2 species;

The first column lists the taxon branch. The second column specifies the used NCBI filter for the assembly level. The third column lists the total number of genomes for a branch. The last column displays the unique number of taxonomic entries at the level of order, family, genus and species.

For DNA-to-protein methods, we used the NCBI non-redundant (nr) protein sequence database that contains entries from GenPept, Swissprot, the Protein Information Resource, the Protein Research Foundation and the Protein Data Bank^60–62^. After under undergoing curation steps, this yielded a protein database containing 433,397,414 sequences, corresponding to 154,116 unique species (see Supplementary for curation details). An overview of the content of this database is available in Table 5. The following DNA-to-protein classifiers were evaluated: Kaiju and MMSeqs2 (see Supplementary for details).

**Table 5:** Protein reference sequences used for DNA-to-protein methods.

Branch	Sequences	# Unique
Archaea	9,370,693	45 orders; 63 families; 215 genera; 3788 species;
Bacteria	389,099,915	261 orders; 662 families; 4090 genera; 81478 species;
Fungi	25,501,044	215 orders; 752 families; 4375 genera; 29135 species;
Protozoa	1,308,088	25 orders; 30 families; 41 genera; 92 species;
Viruses	6,012,833	65 orders; 227 families; 2425 genera; 39622 species;
Eukaryote	1,801,040	1 order; 1 family; 1 genus; 1 species;
Root*	303,801	/

The first column lists the taxon branch. The second column shows the total number of protein sequences per taxon branch. The last column displays the unique number of taxonomic entries at the level of order, family, genus and species.

*Protein sequences at the highest node (either root or cellular organism)

A reference database for DNA-to-marker methods consists of unique clade-specific markers. Since multiple approaches exist to define these markers, each DNA-to-marker tool has its own approach and corresponding database, and a common database for all DNA-to-marker could not be created and the tool-specific databases were used. Although this violated one of our three ground rules for classifier inclusion (i.e., the possibility to create a custom database), we made an exception for DNA-to-marker methods to conceptually evaluate whether this type of classifier potentially could outperform the other two types. The following tools were evaluated: MetaPhlAn3 and mOTUs2 (see Supplementary for details).

Due to the databases being constructed at various time intervals, the volatility of taxonomic IDs within the NCBI Taxonomy database can lead to faulty conclusion when comparing the ground truth of the mock communities with the output of the classifiers. To address this, taxonomic IDs from both the ground truth and classifier outputs were synchronized to correspond to the same time point using Taxonkit v0.13.0 (See Supplementary for details)^63^.

### Performance evaluation

The evaluation was performed in two separate rounds. First, classifiers were evaluated in a ‘per-sample’ manner where each sample was considered separately. Per sample, all performance metrics were calculated for each classifier and an output report was generated^64^. Second, classifiers were evaluated in a ‘per-classifier’ manner. For each classifier, results of all datasets with the R9 chemistry were aggregated and one output report was generated^64^. The R10 dataset of Zymo D6322 was however excluded from this second step since not enough R10 datasets were present for a systematic investigation. Instead, it was specifically compared to its R9 counterpart.

### Per sample evaluation

#### Performance metrics

Classifiers were evaluated by comparing the taxonomic names with the ground truth (i.e., the known organisms of the DMCs). Performance was evaluated at both the genus and species levels. Higher taxonomic ranks were not considered. If classification was possible at genus but not at species level, the taxonomic label was assigned based on the corresponding genus, accompanied by an ‘unclassified’ designation at species level. Commonly used performance metrics to evaluate classifiers include precision, recall and F1^65^. These are based on the number of true positives (TPs), false positives (FPs), and false negatives (FNs). A taxon detected by a classifier was considered a TP if it was present in the DMC. A FP constituted a detected taxon not present in the DMC. A FN constituted a taxon present in the DMC but not detected by a classifier. Precision, recall and F1 were then calculated as follows:

1 $${Precision}=\frac{{TPs}}{{TPs}+{FPs}}$$

2 $${Recall}=\frac{{TPs}}{{TPs}+{FNs}}$$

3 $$F1=\frac{2}{{recal}{l}^{-1}+{precisio}{n}^{-1}}=\frac{2\ast {precision}\ast {recall}}{{precision}+{recall}}=\frac{{TPs}}{{TPs}+\frac{1}{2}({FPs}+{FNs})}$$

Precision describes the percentage of all correctly detected taxa out of all detected taxa. A low precision indicates that many false positive taxa were detected. The recall, also referred to as sensitivity or true positive rate, is the percentage of correctly detected taxa out of all taxa present in the DMC. A low sensitivity indicates that many taxa present in a DMC were not identified. F1 is the harmonic mean between precision and the recall and represents both in one metric. In particular, extreme values of either precision or recall are punished more severely. An F1 score of 1 represents perfect precision and recall, while a score of 0 indicates that the precision and/or recall is 0.

#### Relative abundance estimation

Estimated relative abundances need to be as accurate as possible. However, not every classifier outputs the same kind of relative abundance. DNA-to-DNA methods and DNA-to-protein methods output a classification per read. The relative abundance is calculated as the number of reads classified as a certain taxon divided by the total number of reads. This is also referred to as sequence abundance^66^. Conversely, DNA-to-marker methods represent abundance of certain unique marker regions. This is also referred to as taxonomic abundance^66^. Relative abundances of species in DMCs were only available as sequence abundances and hence had to be converted to taxonomic abundances (see Supplementary).

The L1 distance is then calculated using both the relative abundances of taxa in the ground truth and those detected by the classifier (note that throughout this study, the relative abundance is always considered after omitting the unclassified fractions). If vector **p = **(*p*_1_*, p*_2_*, …, p*_*n*_) and **q = **(*q*_1_*, q*_2_*, …, q*_*n*_) are the *n*-dimensional vectors containing the relative abundances of the taxa from the ground truth and the output of the classifier, respectively, the L1 distance (d_L1_ (**p,q**)) between vector **p** and **q**, equals:

4 $${d}_{L1}\left({\boldsymbol{p}},{\boldsymbol{q}}\right)=\mathop{\sum }\limits_{i=1}^{n}\left|{p}_{i}-{q}_{i}\right|$$

The L1 distance can range between zero (i.e., all TPs are detected with the correct relative abundance) and a maximum of two (i.e., no TPs are detected). The L1 distance was calculated using the relative sequence abundance for DNA-to-DNA and DNA-to-protein methods, and the relative taxonomic abundance for DNA-to-marker methods (see Supplementary). Among the nine R9 datasets, BeiRes_277D lacked information regarding the exact abundances of the species, and was excluded from the L1 distance calculation.

#### Relative abundance threshold filtering

Frequently within taxonomic classification, relative abundance thresholds are enforced for a detected taxon in order to be acknowledged as truly present^28,40,67^. This mitigates the effects of FPs and their penalty on precision, because FPs often turn up with low relative abundances. Since the relative abundance was computed from classified reads, abundance filtering was likewise done exclusively based on classified reads. The effect of a relative abundance filtering on performance was investigated using precision-recall (PR) curves. These display the tradeoff between precision and recall when shifting the relative abundance threshold. By increasing this threshold, precision typically increases but recall decreases. The PR curve can be summarized with the area under the precision-recall curve (AUPRC), which is calculated using the trapezoid rule. A higher AUPRC represents a better model performance. A minimum value of 0 indicates the worst possible performance, while a maximum value of 1 denotes a perfect model where the TPs and FPs can be clearly separated by a specific relative abundance threshold.

### Per classifier evaluation

For every classifier, the results of the R9 datasets were aggregated by calculating the medians of the precision, recall, F1, L1, and AUPRC scores, and depicted in boxplots with values overlayed. Additionally, the medians at every relative abundance threshold were also calculated. Their courses were plotted in a line graph along with the minimum, maximum, 25^th^ percentile (Q1), and 75^th^ percentile (Q3) over all samples. Dotplots were created that showed for all classifiers their median precision and recall on the x-axis and y-axis, respectively, with three different error bars. First, error bars represented the interquartile range (IQR), i.e., Q3-Q1, showing the spread of precision and recall without any abundance filtering. For the second and third, error bars represented the shift of median precision and recall when using a 0.05% and 0.1% relative abundance threshold, respectively, to show the effect of abundance filtering on precision and recall.

## Results

### Performance evaluation of the different taxonomic classifiers

#### DNA-to-DNA methods

**Fig. 1:**
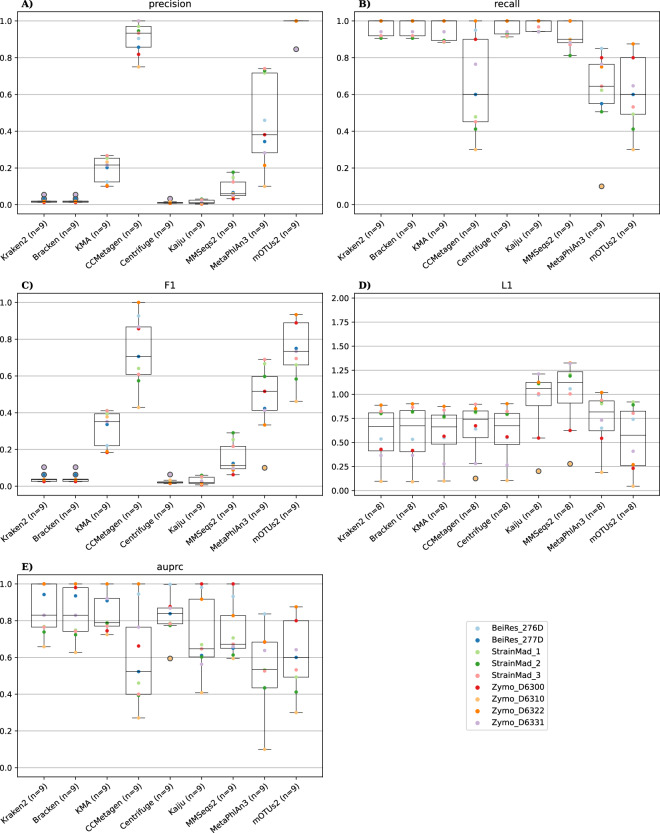
Performance evaluation for the different classifiers aggregated over all DMCs (generated with the R9 technology) at species level. Each subplot represents a performance metric with panels A, B, C, D and E showing precision, recall, F1, L1, and AUPRC, respectively. For each subplot, the y-axis displays the metric value and the x-axis the different classifiers. For every classifier, the metric values of all datasets are summarized in a boxplot with the median value as horizontal line. Individual dots represent specific values for the different DMCs (dots can be superimposed upon each other if the same value was observed). Outliers are denoted by dots enclosed in a black circle. The legend in the lower right panel corresponds to the DMC identifiers presented in Table 1.

The evaluated DNA-to-DNA methods consisted of Kraken2, Bracken, Centrifuge, KMA and CCMetagen. Results described below are at species level (results at genus level are available in the Supplementary). As Bracken and CCMetagen are companion tools building upon the output of Kraken2 and KMA, respectively, their results are separately presented in the next paragraph. Kraken2, Centrifuge and KMA demonstrated low to very low precision for all datasets, i.e., a considerable number of species not present in the DMCs were predicted (Fig. 1A). Although the precision of KMA was low, its median precision was considerably higher (0.216) compared to Kraken2 (0.018) and Centrifuge (0.010). In contrast, all classifiers exhibited high recall, i.e., few false negative species were observed, and the majority of expected species were detected (Fig. 1B). A median recall of 1 was observed for all three classifiers. Moreover, with the exception of the three StrainMad and Zymo_D6331 datasets, the recall of all three classifiers was 1 for the other individual datasets. Although the recall of KMA was slightly lower than Centrifuge and Kraken2, the higher precision of KMA resulted in the highest median F1 score (0.352), followed by Kraken2 (0.035) and lastly Centrifuge (0.019) that introduced much more FPs than Kraken2 (in some samples more than twofold) (Fig. 1C). The L1 distances between all three classifiers were very similar, with a median L1 distance for Centrifuge, KMA, and Kraken2 of 0.667, 0.662, and 0.674, respectively (Fig. 1D).

CCMetagen, a companion tool to KMA that applies post-filtering, had a noteworthy high median precision (0.933). The post-filtering steps removed many FPs, substantially increasing precision compared KMA (0.216), but also unintentionally removed TPs, resulting in a decreasing median recall (0.600) compared to KMA (1). This was most notably observed in datasets with a staggered or logarithmic composition for which the predicted relative abundances of some FPs were close to those of actual TPs, rendering it difficult to separate both. Therefore, CCMetagen performed worst in terms of recall of all DNA-to-DNA methods, but still displayed the highest median F1 score of all DNA-to-DNA classifiers (0.706). CCMetagen had a slightly higher median L1 distance (0.741) compared to Kraken2, KMA, and Centrifuge, because the smaller number of FPs increased the L1 distance but the higher number of FNs increased the L1 distance.

Bracken, a companion tool to Kraken2, re-distributes reads classified at higher taxonomic levels to either the genus or species levels. As Bracken does not introduce or remove new genera or species that were not yet detected by Kraken2, scores such as precision, recall, and F1 will not be altered by Bracken but rather the relative abundances of the detected genera and species are recalculated based on reads assigned to a higher rank. However, the L1 distance differences of Bracken compared to Kraken2 were often very limited. For some samples, such as BeiRes_276, Zymo_D6300, and Zymo_D6310, there was a decrease in L1 distance, and for some samples, such as the three StrainMad, Zymo_D6322 and Zymo_D6331, an increase was observed. This resulted overall in a marginal increase of the median L1 value of Bracken (0.673) compared to Kraken2 (0.667). Bracken, hence, did not exhibit a substantial difference of the relative abundances for the analyzed samples.

#### DNA-to-protein methods

The evaluated DNA-to-protein methods consisted of Kaiju and MMseqs2. Results described below are at species level (results at genus level are available in the Supplementary). Similar to DNA-to-DNA methods, both classifiers displayed only very low precision (Fig. 1A). Kaiju introduced more FPs than MMseqs2, resulting in a lower median precision (0.010) compared to MMseqs2 (0.060). Similar again to DNA-to-DNA methods, both methods displayed very high recall. However, MMseqs2 exhibited more FNs than Kaiju for multiple samples, resulting in lower median recall for MMSeqs2 (0.900) compared to Kaiju (1) (Fig. 1B). The median F1 score of MMseqs2 (0.113) was higher than Kaiju (0.021) (Fig. 1C), due to the pronounced higher precision of MMseqs2 compared to Kaiju. Notwithstanding, the F1 score of MMseqs2 remained substantially lower compared to KMA (0.352). Both DNA-to-protein classifiers generally exhibited worse abundance estimations than DNA-to-DNA classifiers with higher L1 distances, with MMSeqs2 (1.124) exhibiting a worse median L1 distance than Kaiju (1.059) (Fig. 1D).

#### DNA-to-marker methods

The evaluated DNA-to-marker methods consisted of MetaPhlAn3 and mOTUs2. Results described below are at species level (results at genus level are available in the Supplementary). mOTUs2 displayed a substantially higher median precision (1) compared to MetaPhlAn3 (0.381) (Fig. 1A). MetaPhlAn3 displayed a large spread in precision over the different DMCs. The samples that exhibited the lowest precision were those with few species and a staggered or logarithmic composition. Overall, both DNA-to-marker methods consequently performed substantially better in precision compared to DNA-to-DNA and DNA-to-protein methods, excluding CCMetagen (0.933) that achieved a higher precision compared to MetaPhlAn3. However, recall values for both MetaPhlAn3 (0.645) and mOTUs2 (0.600) were also the lowest of all evaluated methods, excluding CCMetagen (Fig. 1B). Because MetaPhlAn3 and mOTUs2 employ different underlying databases that could not be harmonized, the introduction of FNs was however not solely dependent on the classifier’s capability, but also on the presence of the ground truth in their underlying reference databases. Investigation of the underlying databases indicated that mOTUs2 contained fewer taxa from the ground truth in two DMCs and more taxa in one DMC (see Table S1). mOTUs2 had a higher F1 score (0.733) compared to MetaPhlAn3 (0.516) (Fig. 1C), since mOTUs2 had the highest precision and comparable recall to MetaPhlAn3. Consequently, the F1 scores of DNA-to-marker methods were the highest compared to both DNA-to-DNA and DNA-to-protein methods, with again the notable exception of CCMetagen. The L1 distances for MetaPhlAn3 (0.817) and mOTUs2 (0.575) had a substantial difference between each other (Fig. 1D). Notably, mOTUs2 emerged as the classifier with the best L1 distance.

### Relative abundance threshold filtering

#### Area under the precision-recall curve

Overall, DNA-to-DNA and DNA-to-protein methods displayed high to very high recall, but suffered from very low precision, drastically reducing their F1 scores, whereas DNA-to-marker methods displayed medium recall but very high precision, resulting in overall the best F1 scores (Fig. 1). Since classifier performance can be increased by setting an abundance threshold to remove FP predictions, albeit at the cost of increased FNs, PR plots were calculated for all classifiers (see Reports)^64^. The resulting AUPRC values at species level are presented in Fig. 1E (results at genus level are available in the Supplementary). Median AUPRC values were the lowest for the DNA-to-marker methods MetaPhlAn3 (0.533), mOTUs2 (0.600), and the DNA-to-DNA method CCMetagen (0.523). This can likely be explained because recall values of these classifiers were the lowest of all considered categories whereas precision values were the highest, so that further filtering could only reduce recall values with little effect on precision. DNA-to-protein based methods displayed an intermediate effect for both Kaiju (0.647) and MMSeqs2 (0.672), indicating a mildly positive effect of abundance filtering. Lastly, DNA-to-the DNA methods Kraken2 (0.830), Bracken (0.829), Centrifuge (0.838), and KMA (0.789) displayed the highest AUPRC values, excluding CCMetagen. This indicated a marked positive effect of relative abundance threshold filtering for DNA-to-DNA methods with respect to other methods. CCMetagen was an exception because this method performs heavy filtering by default and therefore behaves more similar to DNA-to-marker tools. For all methods, there existed a marked effect of the considered samples on AUPRC values, as expected, since samples with fewer organisms and an even composition exhibited better AUPRC scores. For such samples, it was easier to find a threshold that removed many FP, alleviating the low precision of these methods, without an associated cost of decreasing recall.

#### Effect of abundance filtering on precision, recall and F1

**Fig. 2:**
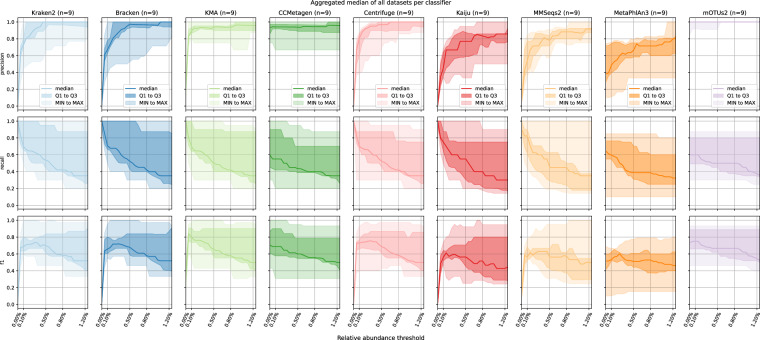
Precision, recall and F1 for the different classifiers when filtering is applied at species level. The first, second and third row represent precision, recall and F1 score, respectively, and each column displays a different classifier. The x-axis of every subplot represents the applied filter threshold for which all species below this threshold were considered as absent, and the y-axis displays the metric value. Each subplot contains three shades of color with the darkest shade showing the median, the medium shade showing the IQR, and the brightest shade showing the minimum/maximum values over all nine R9 DMCs.

As thresholds changed during filtering, precision and recall values also changed. An example is the Zymo_D6300 dataset for Kraken2 (1) and KMA (0.744) with different AUPRC values at species level. Kraken2 became the perfect classifier with a precision and recall of 1 when a filtering threshold of 2.5% was applied. Conversely, whereas KMA exhibited increased precision in the initial filtering thresholds, its precision experienced a rapid decline as the filtering threshold continued increasing due to a FP with a substantial relative abundance. Hence, although AUPRC values indicated DNA-to-DNA methods benefited from increased filtering, finding balanced filtering still requires evaluating precision, recall, and F1 scores at different thresholds to select suitable thresholds for the different classifiers. Figure 2 displays the general trends of precision, recall and F1 at varying thresholds in steps of 0.05% for all classifiers at species level from 0% to 1.20% (results at genus level are available in the Supplementary). As expected, the precision benefitted from increasing relative abundance filtering thresholds, whereas recall was punished, although trends could differ between individual classifiers.

All DNA-to-DNA classifiers had their steepest increase in median precision before a threshold of 0.5%, but the slope of the increase could differ between classifiers, with KMA exhibiting a notably steeper slope compared to Kraken2, Bracken, and Centrifuge. Additionally, both the final maximum median precision and the filtering threshold at which it was reached, could differ between classifiers. The maximum precision of Kraken2 (1), Bracken (1), KMA (0.963) and Centrifuge (1) was reached at a threshold of 0.45%, 1.05%, 0.9% and 0.65%, respectively. However, recall values dropped very fast with increased filtering. At the threshold where DNA-to-DNA classifiers reached their maximum precision, their median recall had decreased drastically. Using F1 scores as a balanced metric for both precision and recall, F1 values experienced the steepest increase up to a threshold of 0.05%, after which the increase slowed down or even decreased, suggesting this to be a well-balanced cutoff for DNA-to-DNA methods. CCMetagen was an outlier for DNA-to-DNA methods as this classifier inherently already performs filtering so that further filtering barely made a difference in precision but decreased recall fairly quickly. Although CCMetagen without filtering scored best in F1 scores compared to other DNA-to-DNA methods, even at very low filtering values, the other DNA-to-DNA methods surpassed CCMetagen, suggesting that the default filters applied to CCMetagen are potentially too strict and should be relaxed. While precision similarly increased for DNA-to-protein methods, its increase was much less steep. Both DNA-to-protein methods had their steepest increase before 0.2% with a similar slope. A maximum median precision of 1 was reached at high filtering thresholds of 2.3% and 1.85% for Kaiju and MMSeqs2, respectively, however similar to DNA-to-DNA methods at substantial costs in recall that were more pronounced for Kaiju. Using F1 scores as a balanced metric for both precision and recall, Kaiju and MMSeqs2 reached their best F1 scores at different filtering thresholds of 0.1% and 0.05%, respectively. Although the steepest increase for MMSeqs2 was before 0.05%, its F1 score still increased at 0.1% without a decrease in recall, suggesting 0.1% to be a well-balanced filtering threshold. Notwithstanding, it appeared that even with tailored filtering thresholds, DNA-to-DNA methods outperformed DNA-to-protein methods because their precision could generally be increased without an as drastic drop in their recall.

Lastly, DNA-to-marker methods similarly displayed increasing precision but with marked differences between mOTUs2 and MetaPhlAn3. mOTUs2 already exhibited a median precision of 1 without any additional filtering, whereas the precision of MetaPhlAn3 benefitted greatly from additional filtering reaching a maximum of 0.917 at a filtering threshold of 2.7%. Recall values declined faster for MetaPhlAn3 than for mOTUs2 with additional filtering. This was reflected in their F1 scores, which suggested filtering thresholds of 0.1% for MetaPhlAn3 and no filtering threshold for mOTUs2.

### Assessment of overall classifier performance

**Fig. 3:**
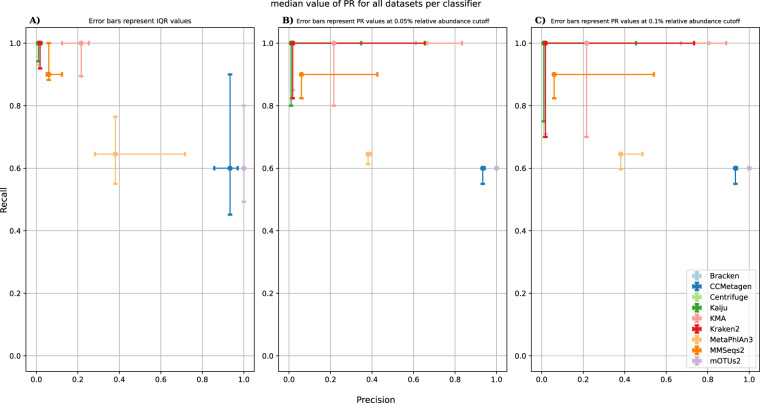
Overall median precision and recall values at species level for the different classifiers. The dots in panel A represent the median precision (x-axis) and recall (y-axis) values for every classifier aggregated over all nine DMCs, while the error bars indicate the extent of the IQR for both the precision and recall. The dots in panels B and C similarly indicate median precision (x-axis) and recall (y-axis) values for every classifier aggregated over all nine R9 DMCs, but with error flags indicating the updated median precision and recall for an abundance filtering threshold of 0.05% and 0.1%, respectively. Classifiers are colored according to the legend on the lower right of plot C. Abbreviations: DMC (Defined mock community); IQR (Interquartile range); PR (Precision recall).

A summary of the performance of all classifiers at species level is presented in Fig. 3A (results at genus level are available in the Supplementary), displaying precision and recall along with their interquartile ranges (based on values obtained over all DMCs) represented as error bars, illustrating a clear distinction between three main groups. The first group contains DNA-to-DNA and DNA-to-protein classifiers, excluding CCMetagen, in the top left corner characterized by high recall but low precision. Within this group, KMA had the best precision. Although its precision exhibited more fluctuation based on its IQR, its lower boundary was still higher than the highest IQR boundary of other classifiers within this group. All classifiers scored a median recall value of 1, except for MMseqs2, although it did reach a recall of 1 for some datasets. The recall IQRs of classifiers, excluding MMSeqs2, were hence similar within this group. The second group consists solely of MetaPhlAn3, which resides in a central position characterized by medium recall and precision. MetaPhlAn3 displayed the highest IQR interval for its precision of all classifiers. Recall was lower compared to the first group, partly explained by missing taxa in the underlying reference database (see Table S1). However, should these missing taxa have been present and correctly detected, MetaPhlAn3 would still have missed more species than the classifiers in the first group (see Table S2) because many species with very low relative abundances were missed. The third group consists of CCMetagen and mOTUs2, residing at the middle right position characterized by high precision but medium recall. Both classifiers exhibited the lowest median recall and largest IQR for recall values among all classifiers. Although mOTUs2 obtained the highest precision close to 1 for all datasets, it experienced the same issue as MetaPhlAn3 with ground truth species being absent in its underlying reference database (see Table S1), having a profound negative impact on recall. However, even if those taxa had been present in the database and detected, the amount of FNs would still have been higher than for other classifiers (see Table S2). CCMetagen, on the other hand, relies on heavy post-filtering of KMA results, increasing precision to very high values but removing too many TPs in the process, especially in datasets with a staggered composition, incurring a heavy penalty in recall.

Figure 3B,C illustrate the effects on precision and recall at species level using filtering thresholds of 0.05% and 0.1% (results at genus level are available in the Supplementary), displaying the effects of filtering as error bars. For the first group, precision increased strongly at 0.05%. Expanding the threshold to 0.1% led to a further increase in precision, albeit to a lesser degree compared to the initial 0.05% threshold. Recall decreased similarly for both thresholds, with the decline being less pronounced for DNA-to-protein methods compared to DNA-to-DNA methods, in agreement with the suggested filtering thresholds of 0.05% and 0.1% for DNA-to-DNA and DNA-to-protein methods (see section Effect of abundance filtering on precision, recall and F1). The second group showed a small increase in precision for a threshold of 0.05% and a bigger increase for a threshold of 0.1%. The associated drops in recall were much less pronounced than for the first group, in agreement with the suggested filtering threshold of 0.1% for MetaPhlAn3 (see section Effect of abundance filtering on precision, recall and F1). Lastly, the third group did not demonstrate any further increases or decreases in both precision and recall when filtering thresholds were increased, in agreement with the suggestion that no filtering should be employed for mOTUs2 and CCMetagen (see section Effect of abundance filtering on precision, recall and F1).

### Evaluation of classification performance using a single ONT R10 DMC

**Fig. 4:**
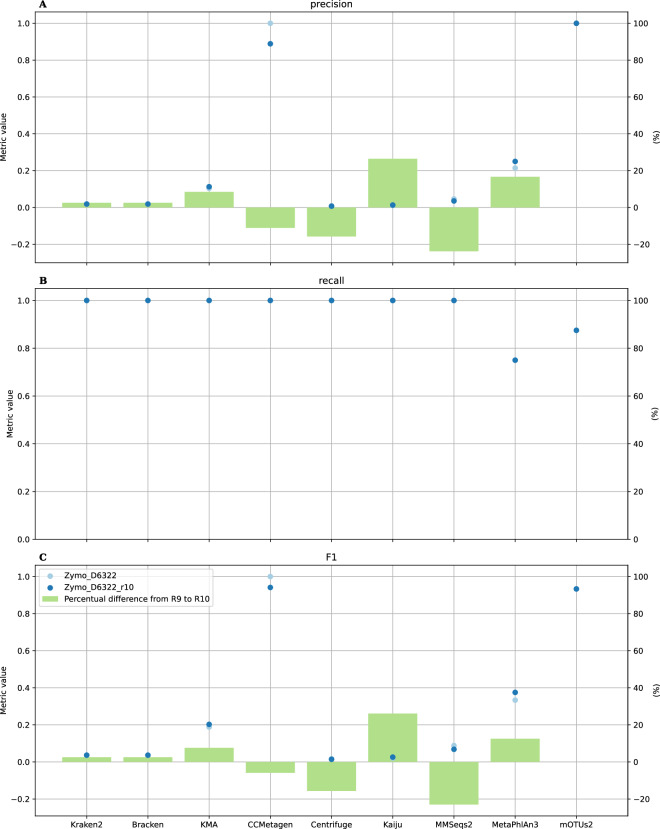
Metric values at species level for the R9 and R10 dataset of Zymo D6322. The dots in panel A, B and C represent the precision, recall and F1 values (left axis), respectively, for every classifier (lower axis) of both the R9 dataset and R10 dataset of the DMC Zymo D6322. Dots can be superimposed upon each other if (nearly) identical values were observed. The bars in each panel present the relative percentage change (right axis) from the R9 to R10 metric value.

Figure 4 presents results for classification performance of all classifiers compared to the R9 and R10 datasets of sample Zymo D6322 at species level (genus level results are available in the Supplementary). For most classifiers, there is no substantial difference in absolute precision when considering both datasets. Only CCMetagen exhibited a notable decline in absolute precision for the R10 dataset, with an absolute decrease of 0.111. However, in relative precision, the R10 dataset showed a substantial decrease for CCMetagen (−11.11%), Centrifuge (−15.78%) and MMseqs2 (−23.79%), whereas a relative precision increase was observed for Kraken2/Bracken (+2.56%), KMA (+8.45%), Kaiju (+26.43%), and MetaPhlAn3 (+16.67%). The precision of mOTUs2 remained the same in both datasets. The notable difference in absolute precision for CCMetagen stems from the low count of FPs in the R9 dataset. Consequently, the introduction of additional FPs in the R10 dataset substantially affected precision for CCMetagen, unlike other classifiers, which already had a higher FP count in the R9 dataset. In contrast, there were no differences in FNs between the R9 and R10 datasets so that the recall for all classifiers remained the same. Consequently, F1 score differences between the R9 and R10 datasets mirrored trends observed for precision with the R10 dataset showing a relative F1 score decrease for CCMetagen (−5.88%), Centrifuge (−15.67%), and MMseqs2 (−22.98%); a relative F1 score increase for Kraken2/Bracken (2.51%), KMA (7.60%), Kaiju (26.09%), and MetaPhlAn3 (12.50%); and the same F1 score for mOTUs2. Note however that the employed R9 dataset of sample Zymo D6322 had a relatively high quality compared to other R9 datasets (see Supplementary Figures S8, S10–S17). This higher quality of the R9 Zymo D6322 dataset was however not an isolated case, as samples Bei Resources HM-277D (Supplementary Figure S11) and Zymo D6331 (Supplementary Figure S17) had comparable read quality distributions to R9 Zymo D6322 (Supplementary Figure S8), demonstrating the variability of nanopore sequencing.

## Discussion

In this study, we extensively benchmarked different taxonomic classifiers on nanopore sequencing data generated on several well-characterized DMCs to provide an overview of the performance of commonly used DNA-to-DNA, DNA-to-protein and DNA-to-marker methods. In particular, we harmonized the underlying reference databases for different methods, and analyzed the largest amount of real DMCs thus far. These DMCs represented a broad range of abundances, taxonomies and application domains, mimicking pathogen, environmental and gut microbiome samples.

DNA-to-DNA methods were characterized by high recall and low precision, excluding CCMetagen that acts as a companion tool to KMA by performing additional filtering^58^. Our benchmarking confirmed CCMetagen to achieve the highest precision and F1 score for all DNA-to-DNA methods, albeit at a high cost in recall. For estimating relative abundances, all DNA-to-DNA methods performed very similarly and were only outperformed by mOTUs2. Bracken acts as a companion tool to Kraken2 by correcting species abundance estimates^55^, but was observed to actually result in slightly worse abundance estimates. Post-filtering classification results greatly increased precision, albeit at a substantial recall cost through missing TP predictions of species present at low abundances, for which we found 0.05% to represent a good trade-off. Since DNA-to-DNA-methods use all genomic information, they had the highest amounts of classified reads, although this also requires *a priori* having full genomes available in contrast to DNA-to-protein and DNA-to-marker methods where only sequence information on proteins and markers, respectively, is required.

DNA-to-protein classifiers also exhibited high recall and low precision. Although recall values were overall comparable to DNA-to-DNA methods (excluding CCMetagen), Kaiju offered over all DMCs the best recall of any of the evaluated classifiers. This effect may be attributable to the underlying databases, as DNA-to-protein classifiers enable building much larger databases since only protein and not full genome sequences are required, allowing to incorporate proteins of species for which no full genomes are available yet. This was for instance observed in the Strain Madness datasets, for which the genomic databases did not contain sequences of some species whereas these were represented in the protein databases. Enforcing relative abundance filtering thresholds indicated that 0.1% represented a well-balanced trade-off between precision and recall. A drawback of DNA-to-protein methods is that many proteins in the database occur in multiple organisms, and hence carry a taxonomic level higher than even the genus so that many reads cannot be classified at species level. Additionally, since only coding regions of reads are classified, many reads remain unclassified due to not containing coding regions. Although we did not benchmark running times, we observed that the requirement to analyze all six possible open reading frames incurred a high computational running cost compared to DNA-to-DNA methods. L1 distances indicated that DNA-to-protein methods resulted in worse abundance estimates compared to DNA-to-DNA methods, which may be attributed to exclusively classifying coding regions, leading to an abundance representation specific to coding sequences.

DNA-to-marker methods exhibited the best precision but also the worst recall of all methods. This was due to a large extent to the absence of some taxa in their underlying reference databases, which have the disadvantage of being built very specifically to a certain tool and therefore being hard to impossible to add new organisms by other users. Post-filtering could therefore not increase the lower recall of DNA-to-marker methods as the TPs were simply not present in the output to begin with. DNA-to-marker methods also had substantially more FNs in datasets with species present at lower relative abundances compared to other methods. Regarding abundance estimates, mOTUs2 emerged as the top-performing classifier of all evaluated classifiers. However, this achievement cannot be primarily attributed to all DNA-to-marker methods, as MetaPhlAn3 demonstrated worse L1 distances compared to DNA-to-DNA methods. It should be noted a newer version of MetaPhlAn3 is available, MetaPhlAn4, which was not available at the time our study was performed. This newer version also includes new species-level genome bins into its underlying reference database, which could affect the recall obtained in our study^68^.

A ‘best classifier’ would require a substantial and consistent performance increase in both recall and precision compared to other classifiers, which was not observed in our study. Instead, our results suggest the choice of classifier should depend on the type of research question(s) and application scope. DNA-to-marker methods provided the highest precision and would therefore be advised if it is crucial that predicted organisms are correct and only limited false predictions are generated. Since the underlying databases may be limited and hard to adapt, they could be less suited for niches where microorganisms are not yet well-represented. If interested in taxonomic abundance rather than sequence abundance, mOTUs2 appears an appropriate choice. Alternatively, certain DNA-to-DNA classifiers like Kraken2 have also been employed alongside targeted amplicon sequencing with a specifically constructed marker database such as the 16S rRNA gene to ascertain taxonomic abundance^69^, but performance evaluation thereof was not within the scope of our study. DNA-to-protein classifiers are interesting for studies where taxa are expected for which protein sequences are already available in sequence databases, but not yet full genome sequences. For other applications, DNA-to-DNA classifiers appear a good choice. Studies focusing on species diversity (e.g., ecological niches) would be expected to benefit of some limited post-filtering to reduce false positive predictions. If detecting all potential organisms is highly relevant, e.g., for clinical applications where a potential pathogen may only be present at very low abundances, post-filtering would be disadvised and screening results for relevant pathogens, followed by additional analyses, such as read mapping, would be recommended in light of their low precision. KMA in particular appears recommended, displaying the highest F1 score, except for CCMetagen and DNA-to-marker methods that are however subject to heavy penalties in recall. Practical considerations could also affect classifier selection. DNA-to-marker methods have the lowest running times, rendering them a good choice to quickly compute results. DNA-to-protein methods have high computational costs and may therefore only be reserved for researchers with access to adequate computational resources. Database creation may also affect classifier selection. KMA database building was a computationally very demanding process. If higher eukaryotes are involved, Kraken2 could be a better choice as we experienced it to be the only classifier that allows building databases containing large and complex eukaryotic genomes, and the low precision could similarly be counteracted with post-filtering and/or other confirmatory assays in case species of low abundance are relevant to detect.

Other benchmarking studies focusing on nanopore sequencing data were conducted by Portik *et al*.^24^ and Marić *et al*.^47^. Portik *et al*. evaluated several short-read methods (Kraken2, Bracken, Centrifuge, mOTUs2 and MetaPhlAn3), long-read methods (MetaMaps, MMSeqs2, MEGAN-LR and BugSeq), and one general method (Sourmash). However, they only evaluated a single DMC twice (each with a different ONT chemistry), compared to nine DMCs in our study, and also did not harmonize the underlying databases of the different classifiers. The majority of those long-read methods were not benchmarked in our study due to our requirements for being open-source, locally installable and customizable. They found that long-read methods generally outperformed short-read methods. Though direct comparison between our study and *Portik et al*. is not possible due to methodological differences, similar trends for the classifiers evaluated in both studies were observed. We also found that classifiers tailored to long reads exhibited better performance. In our study, the recall of MMseqs2 was similar to Portik *et al*. However, their average precision was 2-3 times higher than ours. A potential explanation is that Portik *et al*.‘s calculation of relative abundance relied on all reads rather than just the classified ones, and the application of a default filtering threshold of 0.001%. Applying similar filtering in our analysis consequently increased precision values. KMA was the second long-read classifier considered in our study, and did indeed also outperform short-read methods. With respect to short-read methods, Portik *et al*. similarly observed for Kraken2, Bracken and Centrifuge high recall and low precision, for mOTUs2 almost perfect precision and moderate recall, and for MetaPhlAn3 moderate precision and recall. Lastly, Portik *et al*. found that Bracken did not significantly improve the results of Kraken2, which we also observed. Methodological differences to the study of Marić *et al*. were more profound (e.g., due to employing different definitions for FPs, TPs and FNs). Marić *et al*. divided classifiers into kmer-based methods (Kraken2, Bracken, Centrifuge, CLARK and CLARK-S), mapping-based (MetaMaps, MEGAN-N and deSAMBA), general purpose long-read mappers (Minimap2 and Ram) and tools which use protein databases (Kaiju and MEGAN-P). Although they did create uniform databases for different classifiers in contrast to Portik *et al*., only one real DMC (i.e., not simulated) was evaluated. They found mapping-based methods such as Minimap2 and Ram to outperform kmer-based methods. The latter are however general-purpose read mappers not specifically designed for metagenomic classification, forcing researchers to provide their own scoring schemes and scripts to allow classification. Their results also revealed that tools which used protein databases performed worse than other categories for metrics such as accuracy and abundance estimations, for which the latter aligns with our observations. They observed that kmer-based methods, such as Kraken2 and Centrifuge, introduced many FPs, a phenomenon also seen in our results. They also observed that abundance estimation for kmer-based methods was not on-par with other mapping-based methods, although we found that Kraken2 and Centrifuge exhibited good L1 distances, only outperformed by mOTUs2. Similar to both our study and Portik *et al*., the impact of Bracken on the study conducted by Marić *et al*. was generally negligible.

Our study extends the current knowledge on the performance of metagenomics classification of nanopore sequencing data in several aspects. First, a systematic approach was taken to evaluate different classifiers by generating an extensive uniform report for each sample with performance metrics and figures for easy comparison, and results for different samples were also aggregated to provide a clear and uniform overview of classifier performance based on all DMCs^64^. Researchers interested in better understanding the performance of their favorite classifier on long reads can hence use these reports. Second, a high number of popular classifiers, frequently used for nanopore taxonomic classification based on the literature, was evaluated, including classifiers for which no systematic information on performance was available yet such as KMA and CCMetagen. Third, the used datasets were actually sequenced DMCs without the use of simulated reads. Moreover, to the best of our knowledge, no single other study has evaluated the same quantity of real DMCs. These DMCs encompass varying scenario’s, e.g., both even and staggered species abundance distributions, and mimicking different environments such as the gut and a complex ecosystem hosting numerous species, rendering our benchmarking results more representative. Fourth, the codebase for our performed benchmarking and generating the associated reports, has been made publicly available^64^. This allows other researchers interested in benchmarking their own classifiers to utilize our approach. Fifth, the utilized databases have been harmonized, a practice seldomly observed in other studies, to maximize comparability of different classifiers without introducing unwanted biases from the underlying databases.

We acknowledge the following limitations of our study. First, default parameters were used for all classifiers. With careful parameter tuning, the performance of certain classifiers may potentially still improve substantially. However, in practical applications, classifiers are predominantly utilized with default parameters, as the tuning process demands a substantial amount of time without guaranteed improvement. Notwithstanding, for those interested in parameter tuning, our codebase can serve as a tool to benchmark various parameters by comparing reports generated with different classifier configurations. Second, even though we evaluated the largest amount of DMCs thus far, certain niches, such as ecology of unusual habitats, are not represented in our benchmarking so that our results may not be applicable to those domains. Once such datasets become available, the classifiers can undergo re-evaluation, incorporating their specific characteristics. Third, as the DMCs originate from various studies, procedures for sample collection and processing varied. Differences in DNA extraction, library preparation, sequencing, and other factors could potentially introduce unwanted variability. However, the high amount of DMCs is expected to mitigate these potential sources of variability to some extent. In future work, the impact of these factors could also be assessed with our framework by introducing the same datasets generated by variations in sample processing.

The field of nanopore sequencing is still undergoing rapid evolution, marked by numerous alterations to the underlying flowcells and chemistries. The benchmarking was primarily performed with DMCs that were sequenced using the R9 chemistry, while the recently introduced R10 chemistry is expected to result in higher-quality reads^70^. Moreover, R9 will be phased out in 2024 in favor of R10. As the R10 chemistry is still novel, there is currently a scarcity of R10 reference datasets for conducting rigorous benchmarking, but the higher quality of new ONT chemistries is expected to increase classification performance, especially for DNA-to-protein and k-mer based classifiers^24^. However, in our results, Kraken2’s performance increased while Centrifuge’s declined, both utilizing exact kmers. Similarly, Kaiju’s performance improved while MMseqs2’s decreased, both using translation. Furthermore, an increased performance was observed for KMA and MetaPhlAn3, while a decrease was observed for CCMetagen. Notably, performance differences were solely due to changes in precision whereas recall values remained the same. These findings did hence not yield a definitive conclusion on improvement of performance. Furthermore, the R9 dataset used had above-average quality compared to typical R9 datasets, making the quality difference with the R10 dataset less pronounced. Factors beyond quality, such as read lengths and N50, may also have influenced the comparison of the two chemistries. It hence remains largely unclear what the exact effects, if any, of the new R10 sequencing on the performance of taxonomic classification will be. When more R10 datasets become available, our framework could also allow a more comprehensive comparative analysis of newly released ONT chemistry versions against older versions to estimate improvements in taxonomic classification. Beyond dataset chemistry, as the quantity of public reference ONT datasets grows, our approach could also allow periodic reassessment of classifiers, particularly in niche applications where appropriate reference datasets are currently lacking. In this context, harmonized centrally maintained reference collections of DMCs representing different application domains and ecological niches, sequenced with different sequencing technologies/chemistries would prove to be a major asset. There also exists a need for new algorithmic developments for long-read tools. In contrast to short-read methods, few long-read specific classifiers are currently available. Classifiers need to evolve in tandem with technological advancements to harness the full potential of emerging sequencing platforms. In particular, we urge that these classifiers should be open-source, allow customizable databases, and should be locally installable. These prerequisites offer a multitude of advantages, including interoperability, reproducibility, adaptability, accessibility, security, speed, community support and development. Lastly, harmonization of the underlying reference databases would also allow to more quickly contrast the performance of different classifiers on different sequencing technologies/chemistries.

### Supplementary information

Supplementary File

## Data Availability

The datasets presented in this study originate from other studies and can be found under the run accessions in Table 1. The output reports with all metrics and plots are available on Zenodo (https://zenodo.org/doi/10.5281/zenodo.11371848)^64^.

## Code availability

The source code to perform the analysis and generate the output reports is publicly available on GitHub (https://github.com/BioinformaticsPlatformWIV-ISP/BenchmarkingClassifiers) accompanied by an example dataset showcasing the expected output structure and final output file.
