# Supplementary material for: Benchmarking bacterial taxonomic classification using nanopore metagenomics data of several mock communities
Source: Sci Data. 2024 Aug 10;11:864. doi: 10.1038/s41597-024-03672-8 (PMC11316826; doi:10.1038/s41597-024-03672-8)
Supplement: Supplementary file 1 — Supplementary File [file 41597_2024_3672_MOESM1_ESM.docx]

RESEARCH ARTICLE

Supplementary material

# Text

## Commands

### Kraken2 and Bracken

Kraken2 is a k-mer based classifier which classifies reads based on a lowest common ancestor (LCA) approach. Each node in the taxonomic tree of the database is assigned a weight based on the number of k-mers in the sequence associated with the taxon of the node. A k-mer gets associated with the LCA node of all organisms whose genomes contain that k-mer. Then, all root-to-leaf (RTL) paths are given a score by summing up all the weights of the nodes in the RTL path. The sequence is given the taxon of the leaf from the RTL path with the highest score. Kraken2 v2.1.1 was used with default parameters.

Bracken is a companion program to Kraken2. The reads classified by Kraken2 are converted to abundance estimates while ignoring reads at higher levels, leading to an underestimation of some species. Bracken re-estimates the species abundance by probabilistically re-distributing reads in the taxonomic tree using Bayes’ theorem. Bracken v2.8 was used with the default parameters except for -r 1000.

### Centrifuge

Centrifuge uses the memory-efficient Ferragina-Manzini (FM) index to enable rapid and accurate classification of metagenomic samples. Classification of a read begins with a short exact match that is extended until a mismatch is encountered. The taxonomic ID of the reference sequence to which the match mapped is stored. The algorithm then continues to search for other extended matches in the rest of the reads. Based on the matches, Centrifuge then scores each taxonomic ID using a certain formula. The read is classified as the taxonomic ID with the highest score. Whenever multiple taxonomic IDs have identical scores, an LCA approach is used to reduce the number of assigned taxonomic IDs by traversing the taxonomic tree to higher ranks. The number of final assigned taxonomic IDs can be changed with the parameter -k. This strategy differs from the LCA approach from Kraken2, which always converges to one taxonomic ID. The FM index has two advantages over k-mer based classification. Firstly, the size of the k-mer table is often large and thus requires considerable disk space. Secondly, the use of fixed k-values for k-mers imposes a tradeoff between sensitivity and precision whereas the FM-index enables the search of k-mers of any length. Centrifuge v1.0.4 was used with default parameters except for –k. The parameter defines the maximum number of taxonomic labels for each read. A read can get assigned to multiple species if their assignment score is equal. If the number of assignments is higher than –k , Centrifuge will traverse the taxonomic tree with an LCA approach until less than –k assignments remain. The parameter was set to 1 such that Centrifuge merges some of the assignments into a higher taxonomic rank until one taxonomic label is obtained. This avoids ambiguity when a read is assigned multiple taxonomic groups.

### KMA

KMA is a mapping method which allows for mapping raw reads against a redundant database. The process consists out of 5 steps. Firstly, all reads are trimmed. Secondly, a heuristic k-mer mapping is applied to check whether a given sequence is eligible for further analysis. Thirdly, for all sequences that passed the heuristic mapping, the k-mers are used as seeds to extend the alignment with the Needleman-Wunsch algorithm. Fourthly, in the event of a tie for best matching reference sequences, the ConClave scoring scheme is used which favors reference sequences with more matches to be selected as best match. Lastly, a consensus sequence for a given reference sequence is made out of all the query sequences mapping to the reference. KMA v1.3.28 was used with the parameters –mrs 0.0, -bcNano, -bc 0.7, -ef, -a, -mem_mode, -1t1 and –matrix, as these parameters are recommended in the manual for genome mapping with nanopore reads. Reads were assigned the taxonomic ID of the reference sequence to which they mapped.

### CCMetagen

CCMetagen is a metagenomic classification pipeline around KMA which offers additional processing and filtering of the results, including two noteworthy steps. Firstly, reads are only classified if their consensus sequence (constructed by KMA) has a minimum sequencing depth, coverage and query identity to the reference sequence. By imposing these minima, the chance of false positives is reduced. Secondly, the lowest taxonomic rank that can be assigned to a consensus sequence is determined by the sequence similarity between the consensus and the reference sequence. The default similarity threshold for each taxonomic rank is pre-defined based on large-scale analysis. CCMetagen v1.4.1 was used with all parameters except -r RefSeq and -m text.

### Kaiju

Kaiju uses a modified version of the backwards search algorithm in the Burrows–Wheeler transform allowing querying large sets of sequencing reads. Before the DNA sequences can be mapped against the protein reference database, they are translated into the six possible reading frames and split in amino acid fragments at stop codons. Kaiju has two search modes to classify a read: exact and greedy. For the greedy search, the longest maximum exact match (MEM) against the reference database is searched for amongst all the fragments. The read is assigned to the taxon of the reference with the longest MEM. If there are multiple MEMs, the taxon is determined according to their LCA. The greedy search mode also starts with a (smaller) MEM but is used as seed to extend alignment from by allowing substitutions. Each fragment is scored using a BLOSUM62 matrix and the fragment with the highest score is used to classify the read. If there are fragments with an equal score, the taxon is determined according to their LCA. The greedy search mode of Kaiju v1.9.0 was used with default parameters.

### MMSeqs2

MMSeqs2 consists out of three steps. Firstly, all sequences are translated according to the six possible reading frames. Secondly, all fragments unlikely to find a taxonomic hit in later stages are rejected, i.e. fragments with fewer than three consecutive similar k-mer matches. Thirdly, a novel LCA approach called ‘approximate 2bLCA’ is used to classify the reads. The workflow *easy-taxonomy* of MMSeqs2 v13.45111 was used with default parameters.

### MetaPhlAn3

The database release *mpa_v31_CHOCOPhlAn_201901* of MetaPhlAn3 contains roughly 1.1 million unique clade-specific marker genes identified from ~99,500 bacterial and archaeal and ~500 eukaryotic reference genomes. MetaPhlAn3 maps the raw reads to the marker gene database using bowtie2 and estimates the coverage of each marker. Per clade, the average coverage is calculated using the coverages of the marker genes belonging to that clade. The relative taxonomic abundance is obtained by normalizing the clade’s coverage across all detected clades. MetaPhlAn v3.0.14 was used alongside bowtie v2.4.1. Default parameters were used except --bt2_ps 'sensitive-local' and --unknown_estimation. The first parameter sets the alignment mode to local as the default is global which did not yield any results. The second parameter is added so the unknown proportion is also in the output.

### mOTUs2

mOTUs2 uses phylogenetic marker gene (MG)-based operational taxonomic units (mOTUs) to classify reads. Reference genomes are grouped into species-level clusters and the MG sequences of the reference genomes are grouped based on their species-level affiliation into marker gene clusters (MGCs). An mOTU is then a group of MGCs of different MGs. These mOTUs can be either derived from reference genomes (ref-mOTUs) or from metagenomic samples (meta-mOTUs). We used database v2.6.0 which contains 11,915 ref-mOTUs based on 40 universal single-copy marker genes from more than 25,000 reference genomes. The workflow of mOTUs2 consists out of three steps. Firstly, all raw reads are mapped to MGs in the database using BWA-MEM. Secondly, the read abundance of each MGC is estimated based on the mappings of the MGs in the MGC. Lastly, the abundances of the mOTUs are calculated by taking the median of their respective MGCs’ abundance. mOTUs2 v2.6 was used alongside bwa v0.7.17 and samtools v1.17. Originally, mOTUs2 was developed to only handle short reads. However, the developers later added the ability to classify long reads by cutting the short reads into smaller pieces. Thus, the long reads were chopped into smaller reads with the command motus prep_long. Afterwards, motus profile was used with parameters –p, -u, -e and –q.

## Employed reference database

### Genomic database

On 24 February 2023, genomes from the taxonomic branches archaea, bacteria, fungi, human, protozoa and viruses were downloaded. The assembly presentation was filtered on ‘full’ for genomes. The assembly level was set to ‘complete’ for the bacteria and viruses. To increase the quantity of genomes, the assembly level for archaea, fungi, and protozoa was extended to include 'scaffold' and 'contig', since only few complete genomes were otherwise available. Additionally, since the employed database was used across various research projects not in the scope of this study, nine custom genomes were added, of which four bacteria (GCF_000238355.1, GCF_000260835.1, GCF_018967645.1, GCF_900236745.1), four fungi (GCA_900893395.1, GCA_025331445.1, GCA_028975445.1, GCA_028975465.1) and one unicellular alga (GCA_900893395.1).

### Protein database

In the NCBI nr protein database, all identical sequences are merged into one entry. Consequently, each sequence in the database is unique but can originate from distinct organisms. For consistency, the same taxonomic filtering was applied as in the RefSeq database, i.e., proteins belonging to the taxonomic branches archaea, bacteria, fungi, human, protozoa, and viruses. On 5 September 2022, proteins from those respective taxonomic branches were extracted using blastdbcmd/2.13.0^1^. Protozoa do not correspond to a separate branch in the NCBI taxonomic database, although it is a separate taxonomic branch in RefSeq. A set of taxonomic IDs derived from the protozoan assemblies in RefSeq was therefore used. A Lowest Common Ancestor (LCA) approach was used to assign one taxonomic ID to each protein sequence since entries in the nr protein database could originate from different organisms. As a final processing step, all proteins with non-canonical amino acids (i.e., B, J, O, U, Z, and X) were removed from the database because not all classifiers could handle these non-canonical amino acids.

### Taxonomic IDs

During classification, each sequence read gets assigned a taxonomic ID dependent on matching in the reference database. These taxonomic IDs originate from the NCBI Taxonomy database and are subject to continuous change as new taxa are added^2^. Consequently, taxonomic IDs may undergo merging or deletion. Likewise, taxonomic names may change due to synonyms, misspellings, etc. The volatility of taxonomic IDs and names poses a problem when comparing the ground truth of the mock communities with the output of the different classifiers. Because the output of a classifier depends on the taxonomy files used to build the classifier’s database and because the taxonomy files change over time, comparing the ground truth and the output of the classifier can lead to incorrect conclusions (see Figure S1). However, NCBI tracks merged and deleted taxonomic IDs alongside the current taxonomic IDs and names. This information is available in the taxonomy dump files on the NCBI ftp server. Using the same taxonomy dump files to update the taxonomic IDs obtained on different dates solves the problem of merging taxonomic IDs. Additionally, translating the updated taxonomic IDs into taxonomic names (using again the same taxonomy dump files) ensures that the same taxonomic IDs result in the same taxonomic names. Taxonkit v0.13.0^3^ was used to update the taxonomic IDs of the ground truth and classifiers’ output. Taxonomic IDs that were merged were updated to their new taxonomic ID, and deleted taxonomic IDs were flagged. Afterwards, the taxonomic IDs were converted to their genus and species name. In case a certain taxonomic ID had a species rank, but not genus rank, the taxonomic name of the genus was converted to ‘missing_genus’. These names were used to compare the output of the classifiers to the ground truth.

## Metric formulas

|  | $\frac{S_{i}}{T_{i}}=\frac{\sum_{i=1}^{n} \frac{R_{i}}{(L_{i}P_{i})}}{\sum_{i=1}^{n} R_{i}}*L_{i}P_{i}$ |  |
| --- | --- | --- |

where S_i_ is the sequence abundance, T_i_ is the taxonomic abundance, R_i_ is the number of reads, L_i_ is the genome length, and P_i_ is the ploidy of taxon i. Because L_i_ and P_i_ vary across different taxa, S_i_ and T_i_ are not connected by any universal or sample-independent algebraic relation. However, the taxa in the DMCs are known. Consequently, L_i_ can be determined through the representative genome on NCBI for every taxon. Regarding ploidy Pi, a value of 1 was used for prokaryotes and a value of 2 for eukaryotes.

## Performance evaluation of the different taxonomic classifiers at genus level

### DNA-to-DNA methods

At genus level, many of the same trends as observed at species level were apparent (Figure S2). The median precision of KMA (0.486) increased substantially while the median precision of Kraken2 (0.048) and Centrifuge (0.025) stayed low. The median recall for Kraken2, KMA and Centrifuge for all datasets was 1 except for the three StrainMad and Zymo_D6331 datasets for which KMA scored less than Kraken2 and Centrifuge. Nevertheless, the median F1 score of KMA (0.654) was much higher compared to Kraken2 (0.092) and Centrifuge (0.049). The median L1 distances at genus level were lower than at species level, but again very similar with median values of 0.554, 0.564 and 0.572 for Kraken2, KMA and Centrifuge, respectively. CCMetagen’s precision (0.971) and recall (0.706) increased, still being the highest and lowest, respectively, resulting in the highest F1 score (0.782). Similar to the species level, Bracken did not substantially change the relative abundance estimates of Kraken2.

### DNA-to-protein methods

At genus level, the same trends as at species level were apparent (Figure S2). The precision of MMSeqs2 (0.125) was still higher than Kaiju’s precision (0.036). The difference in recall between Kaiju and MMSeqs2 was less pronounced. While Kaiju retained a median recall of 1, the median recall of MMSeqs2 also increased to 1. Furthermore, MMSeqs2 had only two datasets with a slightly lower recall than Kaiju, compared to five datasets at species level. The difference in median F1 score increased between Kaiju (0.069) and MMSeqs2 (0.222). The median L1 distance of Kaiju (0.689) approached that of DNA-to-DNA classifiers while the L1 distance of MMSeqs2 (0.825) remained the highest.

### DNA-to-marker methods

At genus level, the median precision of MetaPhlAn3 (0.556) increased. The median precision of mOTUs2 remained 1, but surprisingly the precision of two datasets decreased. This decline can be attributed to certain mOTUs encompassing multiple species. In such instances, these mOTUs are labeled as 'unclassified' at species level but are still included at genus level, akin to an lowest common ancestor approach. Consequently, this could potentially lead to false positives at the genus level while maintaining precision at the species level, resulting in an overall lower precision at the genus level. The median recall of both classifiers increased, but the difference between MetaPhlAn3 (0.702) and mOTUs2 (0.647)became more pronounced. The F1 score of MetaPhlAn3 (0.684) increased substantially compared to the marginal increase of mOTUs2 (0.759). Noteworthy, while mOTUs2 had the highest median F1 score of all classifiers at the species level, CCMetagen’ s median F1 score was the highest at genus level. The difference in the median L1 distance was less pronounced with for both MetaPhlAn3 (0.652) and for mOTUs2 (0.524).

### Area under the precision-recall curve

At genus level, all classifiers had an increased median AUPRC with the exception of a marginal decrease for mOTUs2 compared to the species level (Figure S2). Both DNA-to-marker methods, MetaPhlAn3 (0.687) and mOTUS2 (0.596), still had the lowest median AUPRC. Remarkably, DNA-to-protein methods, Kaiju (0.940) and MMSeqs2 (0.942), experienced the highest increase in AUPRC and as a consequence had higher AUPRC scores than the other classifiers. For DNA-to-DNA methods, the median AUPRC for Kraken2 (0.919), Bracken (0.919), KMA (0.935), Centrifuge (0.890) and CCMetagen (0.654) increased. While CCMetagen’ s AUPRC value was the lowest at species level, the median AUPRC of mOTUs2 became the lowest at genus level.

### Effect of abundance filtering on precision, recall and F1

At genus level, the trends of precision, recall and F1 at varying thresholds between the different classifiers were similar as at species level but less pronounced (Figure S5). Additionally, the spread of the IQRs and the minimum/maximum values were lower. The maximum precision of DNA-to-DNA methods, including CCMetagen, was reached very quickly at a low threshold except for Centrifuge, which reached this at a higher threshold. KMA and CCMetagen were able to reach a maximum precision of 1, which they could not achieve at species level. The slope of the recall followed a similar course as at species level with the steepest descent before a threshold of 0.1%. Because the maximum precision was higher and the recall followed the same pace, a higher maximum F1 score was reached with, and for Kraken2 (0.846), Bracken (0.838), KMA (0.903) and Centrifuge (0.843). As at species level, a filtering threshold of 0.05% appeared well-balanced for these methods. For CCMetagen, the highest F1 score was reached without filtering. Although the increase of precision for DNA-to-protein methods at genus level was still slower than DNA-to-DNA methods, their maximum precision was reached faster than at species level. Again, the slope of the recall followed a similar course as at species level with the steepest descent before a threshold of 0.15%. Consequently, similar to DNA-to-DNA methods, their maximum median F1 score was higher and reached faster. Kaiju (0.804) and MMSeqs2 (0.800) reached a maximum median F1 score at 0.2% and 0.05%, respectively. Therefore, less strict filtering than at species level was required with 0.05% appearing a well-balanced cutoff. Lastly, for both DNA-to-markers methods, their maximum F1 score was also higher and reached faster with 0.725 at 0.2% for MetaPhlAn3, and 0.759 without filtering for mOTUs2. Since the F1 score for MetaPhlAn3 decreased, and the F1 score of mOTUs2 remained constant before a filtering threshold of 0.1%, it appeared no additional filtering is recommended for these methods at genus level.

### Assessment of overall classifier performance

A summary of precision and recall of all classifiers at genus level is displayed in Figure S6A. The same three groups as at species level were apparent but less pronounced due to a substantial improvement of KMA’s precision, which was comparable to MetaPhlAn3’s precision. For the first group, recall values were less spread out and MMSeqs2 reached a median recall of 1. The second group, i.e., MetaPhlAn3, had a notably improved precision and a higher IQR for recall. The third group did not change much, except for a slightly higher recall for both CCMetagen and mOTUs2, resulting in CCMetagen having a higher median recall than mOTUs2. Figures S6B and S6C show the transition of the recall and precision as error bars after applying filter thresholds of 0.05% and 0.1%, respectively. The first group’s recall decreased less and its precision improved more than at species level. For the second group, the change in precision and recall was very similar to the change at species level with only an increase in precision at a threshold of 0.1% and a small decrease in recall for both thresholds. For the third group, the filtering hardly made a difference, except for a small improvement of the median precision and a decrease in recall for CCMetagen. Coupled with the filtering effect on F1 values (Figure S5), recommended threshold values of 0.05% were therefore advised for the first group, and no filtering for the second and third group.

## Evaluation of classification performance using a single ONT R10 DMC at genus level

Figure S7 presents results for classification performance of all classifiers compared to the R9 and R10 datasets of sample Zymo D6322 at genus level. At species level, only CCMetagen showed a substantial shift in absolute precision, whereas at genus level KMA (0.072), CCMetagen (-0.111) and MetaPhlan3 (0.061) all displayed notable change. In relative precision, the difference at genus level between the R9 and R10 dataset followed a similar trajectory as observed at species level, albeit with a larger magnitude of change. The relative precision decreased for CCMetagen (-11.11%), Centrifuge (-23.50%) and MMseqs2 (-17.92%); and increased for Kraken2/Bracken (+5.26%), KMA (+26.09%), Kaiju (+25.14%), and MetaPhlAn3 (+19.05%). The precision of mOTUs2 remained the same in both datasets. In contrast, there were no differences in FNs between the R9 and R10 datasets so that the recall for all classifiers remained the same. Consequently, F1 score differences between the R9 and R10 datasets mirrored trends observed for precision with the R10 dataset showing a relative F1 score decrease for CCMetagen (-5.88%), Centrifuge (-23.14%), and MMseqs2 (-16.67%); a relative F1 score increase for Kraken2/Bracken (4.85%), KMA (19.36%), Kaiju (24.06%), and MetaPhlAn3 (13.79%); and the same F1 score for mOTUs2.

# Figures

## Figure S1


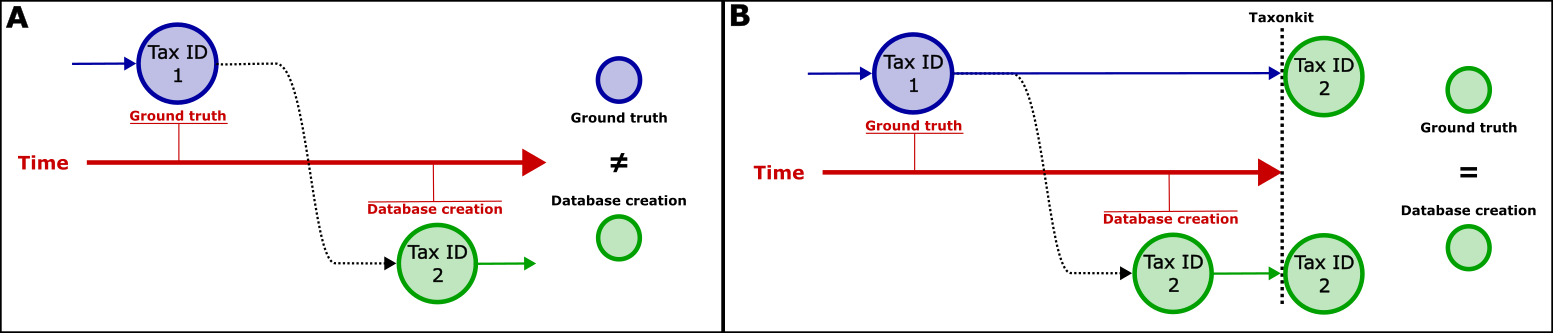


**Figure S1: Comparing two tax IDs obtained at different dates can result in a faulty conclusion due to changes in taxonomic ID.** A) Tax ID 1 from the ground truth and tax ID 2 from the reference database point to the same species but have a different taxonomic ID due to being collected at a different point in time. Comparing the two tax IDs can therefore lead to the faulty conclusion of being different species. B) Because NCBI keeps track of merged and deleted tax IDs, Taxonkit can be used to update tax IDs so the ground truth and the output of the classifier will be the same if the species/genus is the same.

## Figure S2


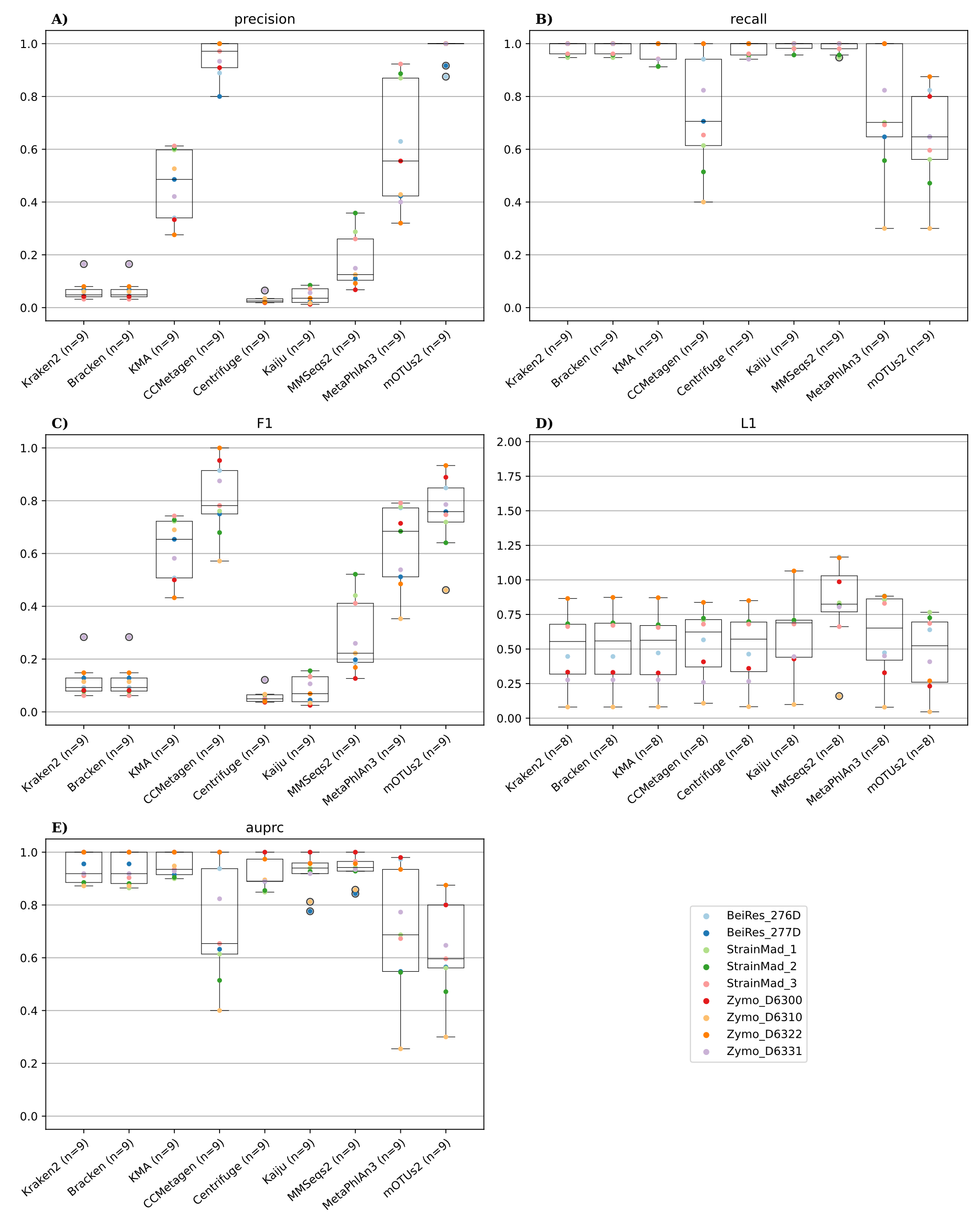


**Figure S2: Performance evaluation for the different classifiers aggregated over all DMCs at genus level.** Each subplot represents a performance metric with panels a, b, c, d and e showing precision, recall, F1, L1, and AUPRC, respectively. For each subplot, the y-axis displays the metric value and the x-axis the different classifiers. For every classifier, the metric values of all datasets are summarized in a boxplot with the median value as horizontal line, upon which superimposed individual dots represent specific values for the different DMCs (dots can be superimposed upon each other if the same value was observed). Outliers are denoted by dots enclosed in a black circle. The legend in the lower right panel corresponds to the DMC identifiers presented in Table 1.

## Figure S3


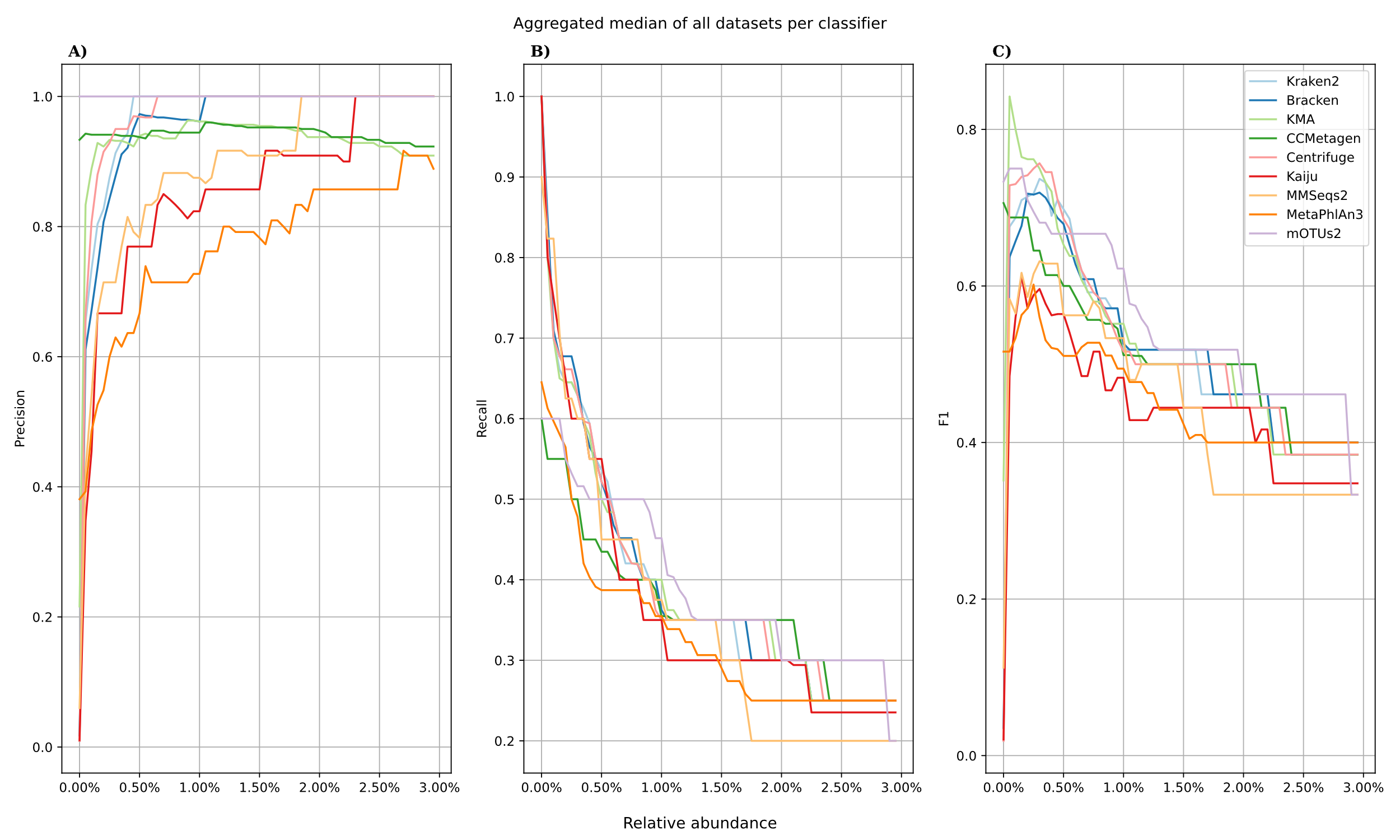


**Figure S3: Precision, recall and F1 when filtering is applied at species level.** The first, second and third plot show the median precision, recall and F1 scores, respectively, for the different classifier over various threshold filters.

## Figure S4


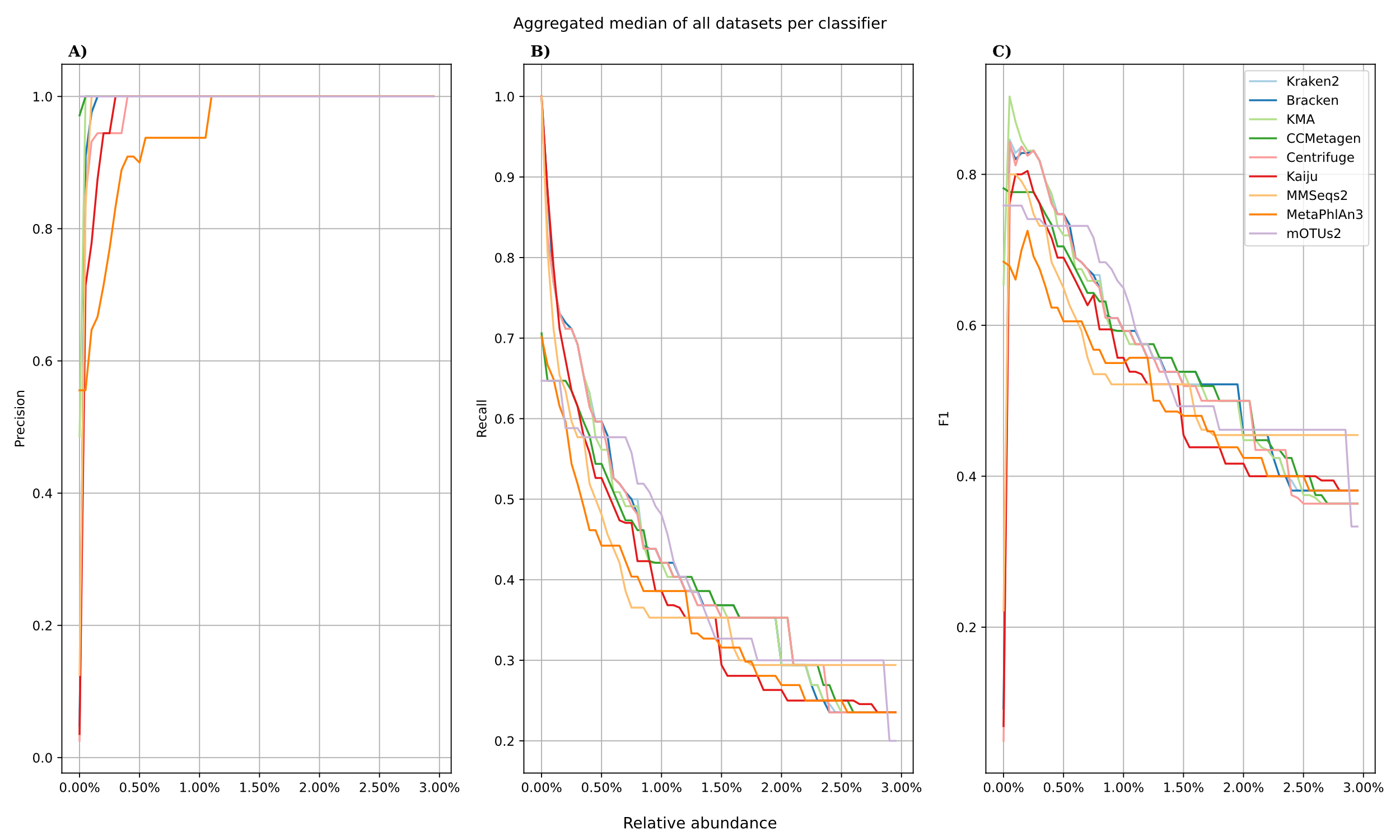


**Figure S4: Precision, recall and F1 when filtering is applied at genus level.** The first, second and third plot show the median precision, recall and F1 scores, respectively, for the different classifier over various threshold filters.

## Figure S5


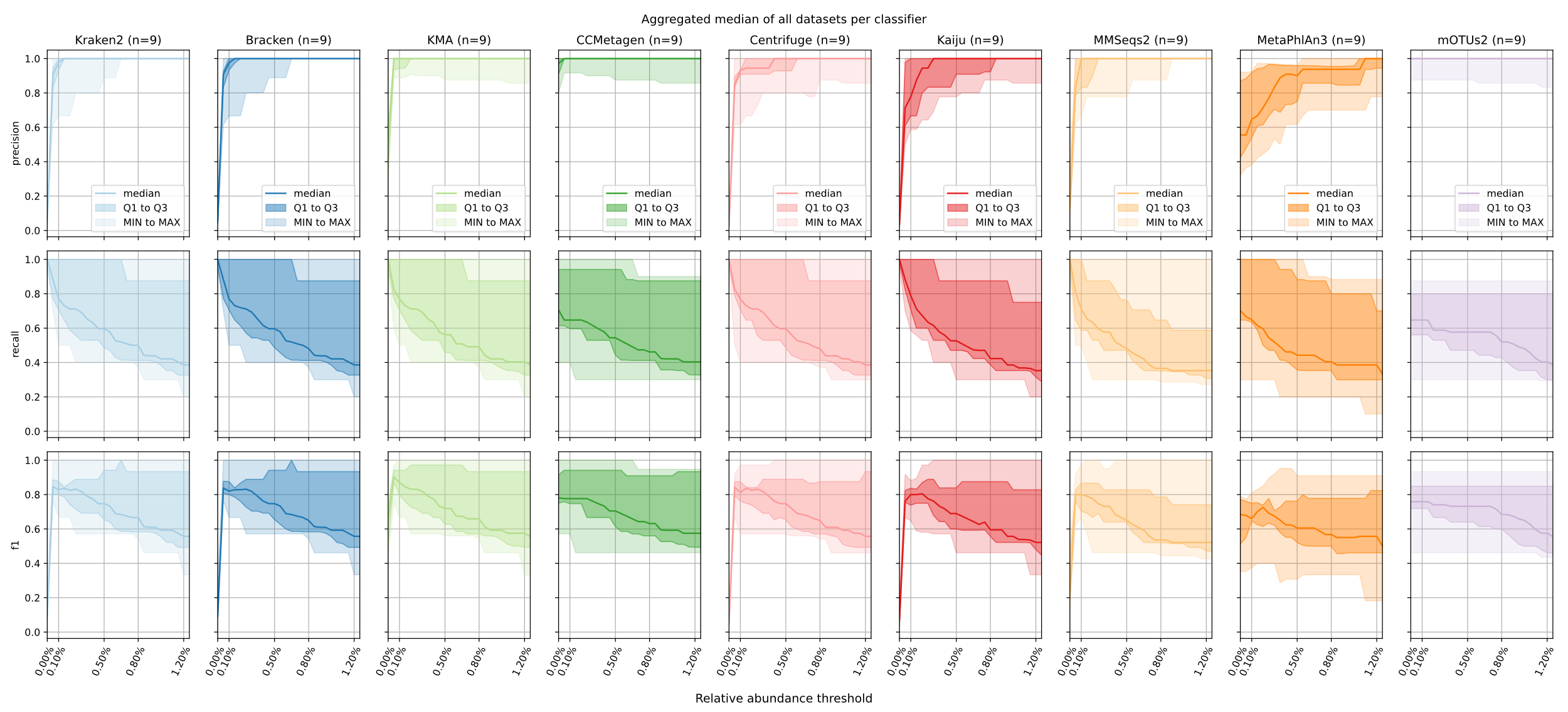
**Figure S5: Precision, recall and F1 for the different classifiers when filtering is applied at genus level.** The first, second and third row represent precision, recall and F1 score, respectively, and each column displays a different classifier. The x-axis of every subplot represents the applied filter threshold for which all species below this threshold were considered as a negative, and the y-axis displays the metric value. Each subplot contains three shades of color with the darkest shade showing the median, the medium shade showing the IQR, and the brightest shade showing the minimum/maximum values over all DMCs.

## Figure S6


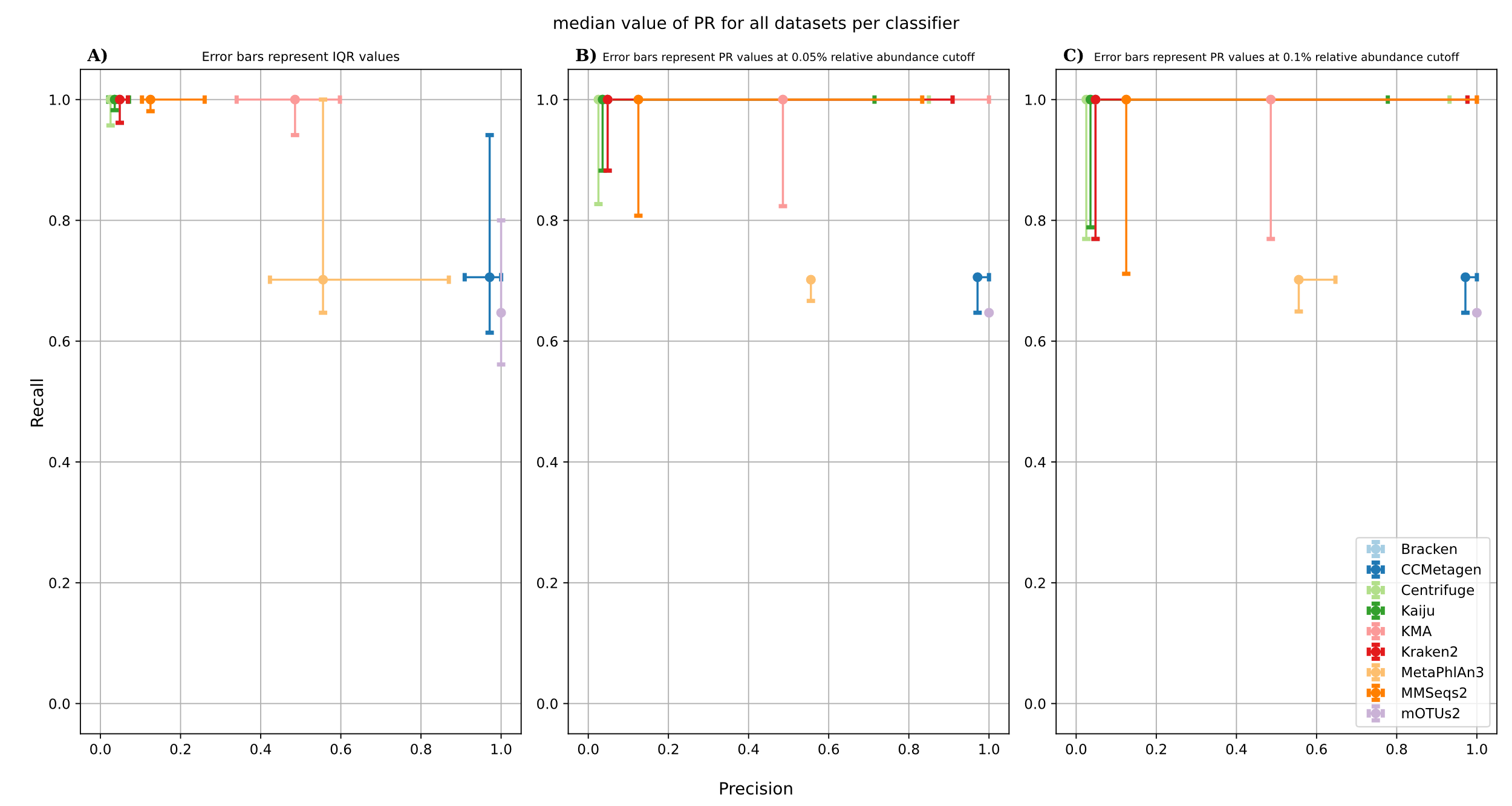


**Figure S6: Overall median precision and recall values at genus level for the different classifiers.** The dots in panel a represent the median precision (x-axis) and recall (y-axis) values for every classifier aggregated over all DMCs, while the error bars indicate the extent of the IQR for both the precision and the recall. The dots in panels b and c similarly indicate median precision (x-axis) and recall (y-axis) values for every classifier aggregated over all DMCs, but with error flags indicating the updated median precision and recall for an abundance filtering threshold of 0.05% and 0.1%, respectively.

## Figure S7


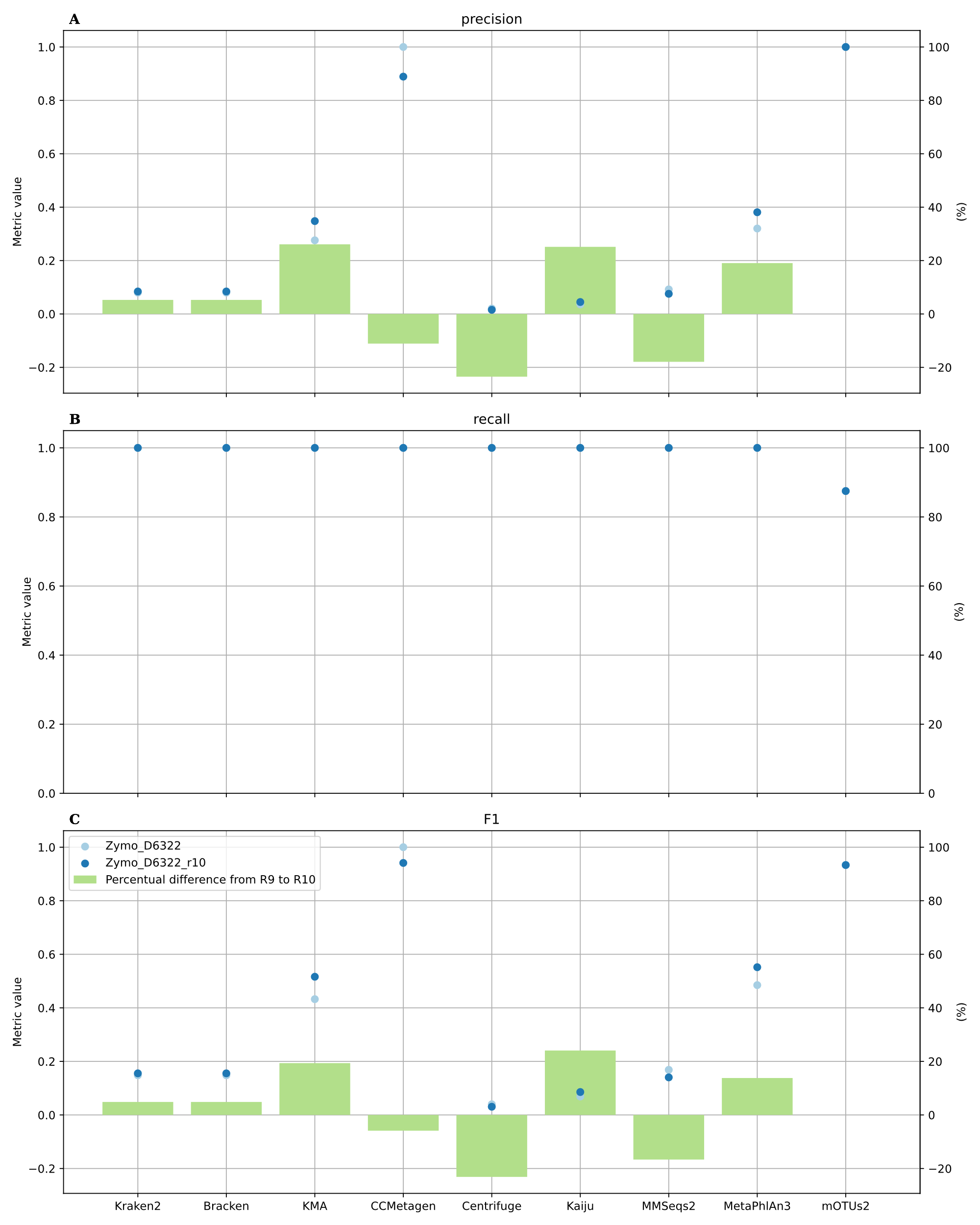


**Figure S7: Precision, recall and F1 values at genus level for the R9 and R10 dataset of Zymo D6322.** The dots in panel A, B and C represent the precision, recall and F1 values (left axis), respectively, for every classifier of both the R9 dataset and R10 dataset of the DMC Zymo D6322. Dots can be superimposed upon each other if (nearly) identical values were observed. The bars in each panel present the percentage change (right axis) from the R9 to R10 metric value.

## Figure S8


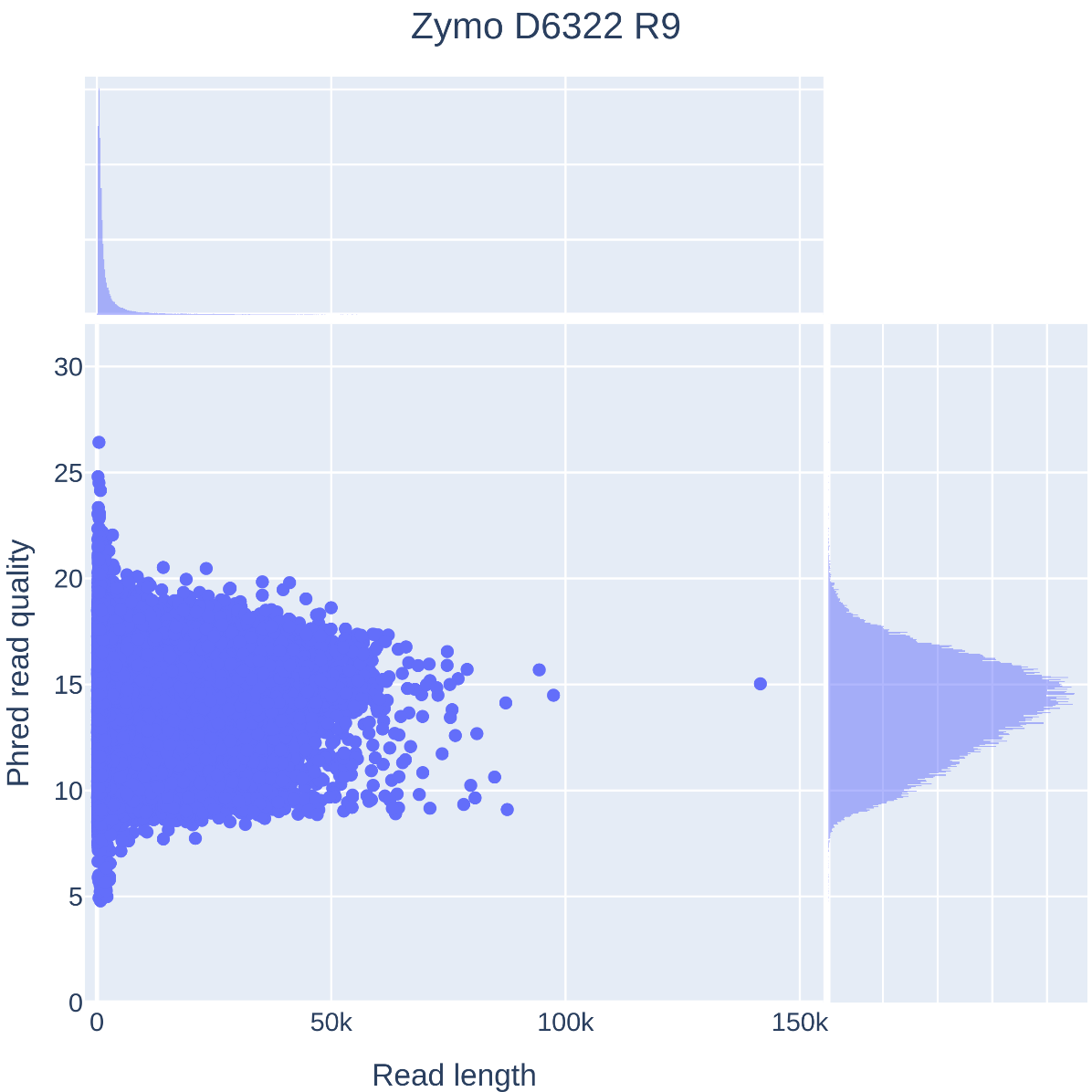


Figure S8: A scatter plot of the reads for Zymo D6322 R9. The x-axis represents the length while the y-axis represents the mean quality of each read. The histograms of the length and quality are shown at the top and right side, respectively. A random subsample of 100,000 reads is plotted.

## Figure S9


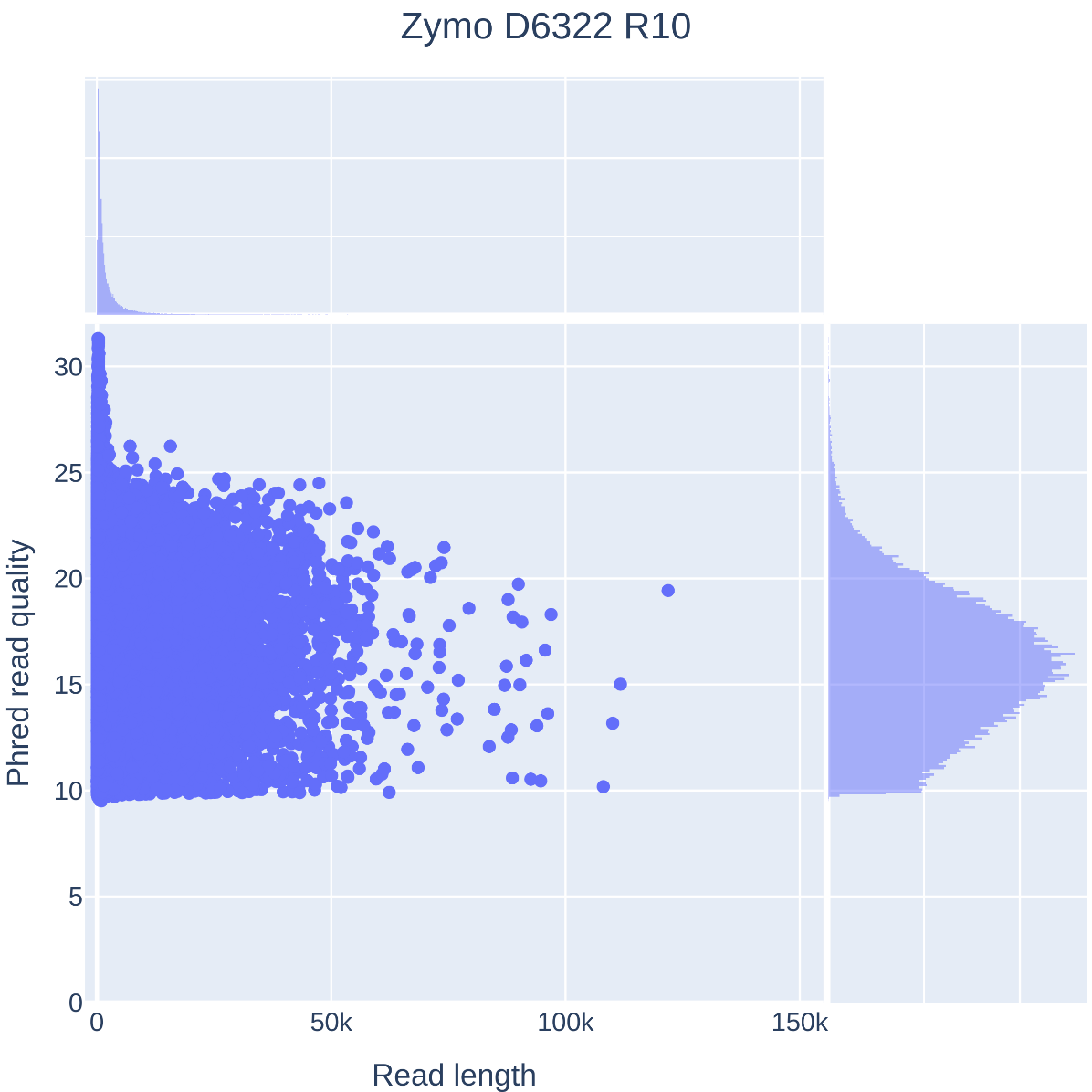


Figure S9: A scatter plot of the reads for Zymo D6322 R10. The x-axis represents the length while the y-axis represents the mean quality of each read. The histograms of the length and quality are shown at the top and right side, respectively. A random subsample of 100,000 reads is plotted.

## Figure S10


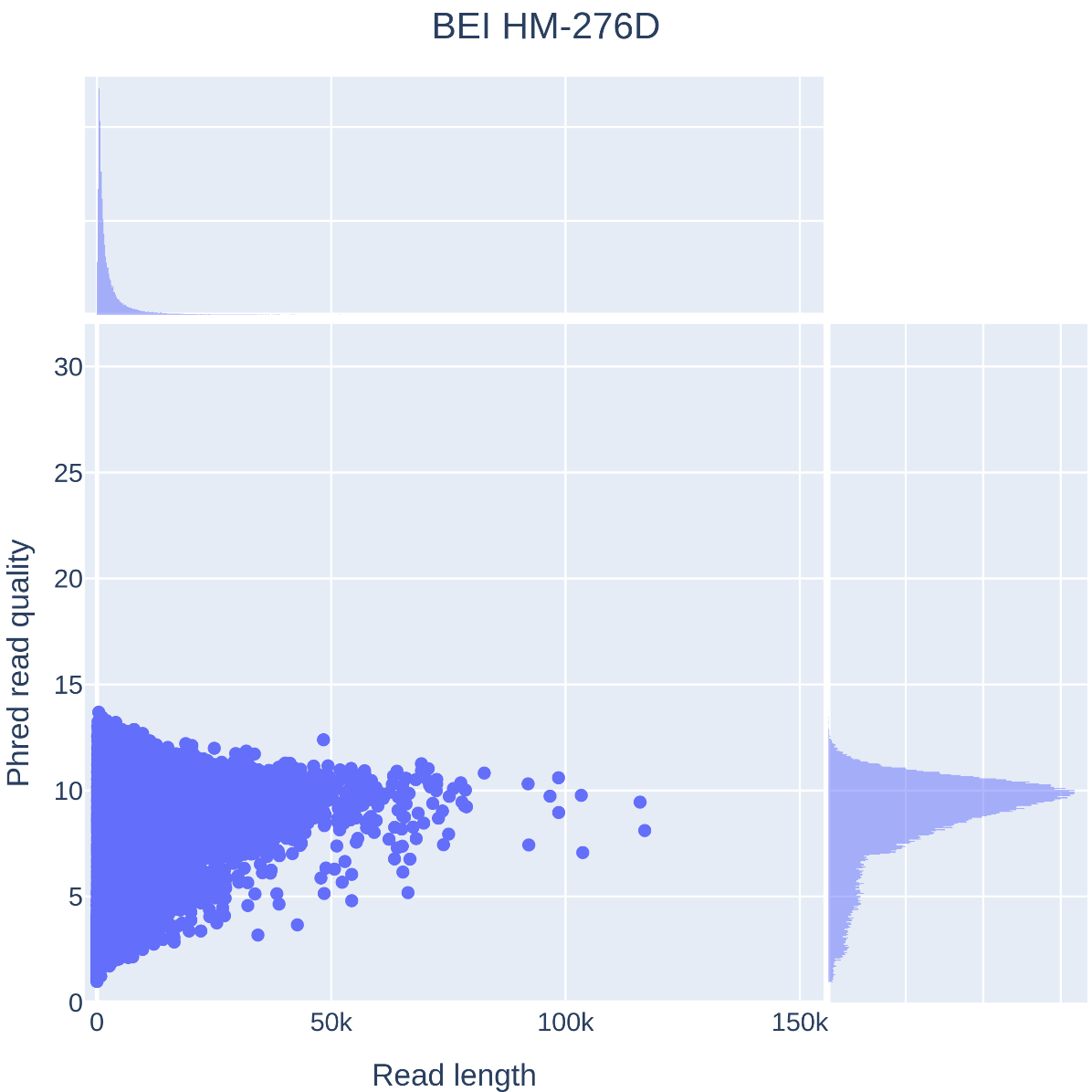


Figure S10: A scatter plot of the reads for BEI HM-276D. The x-axis represents the length while the y-axis represents the mean quality of each read. The histograms of the length and quality are shown at the top and right side, respectively. A random subsample of 100,000 reads is plotted.

## Figure S11


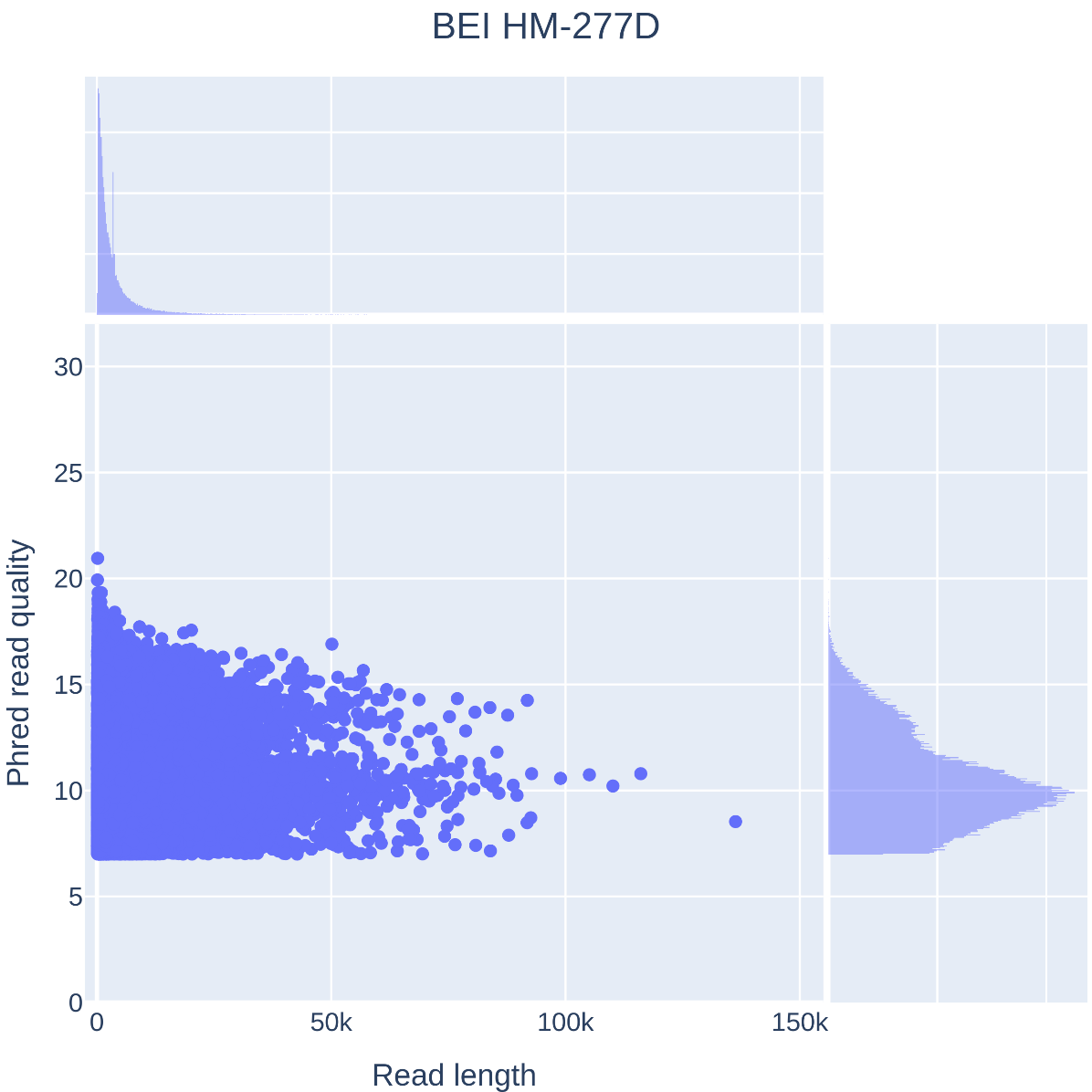


Figure S11: A scatter plot of the reads for BEI HM-277D. The x-axis represents the length while the y-axis represents the mean quality of each read. The histograms of the length and quality are shown at the top and right side, respectively. A random subsample of 100,000 reads is plotted.

## Figure S12


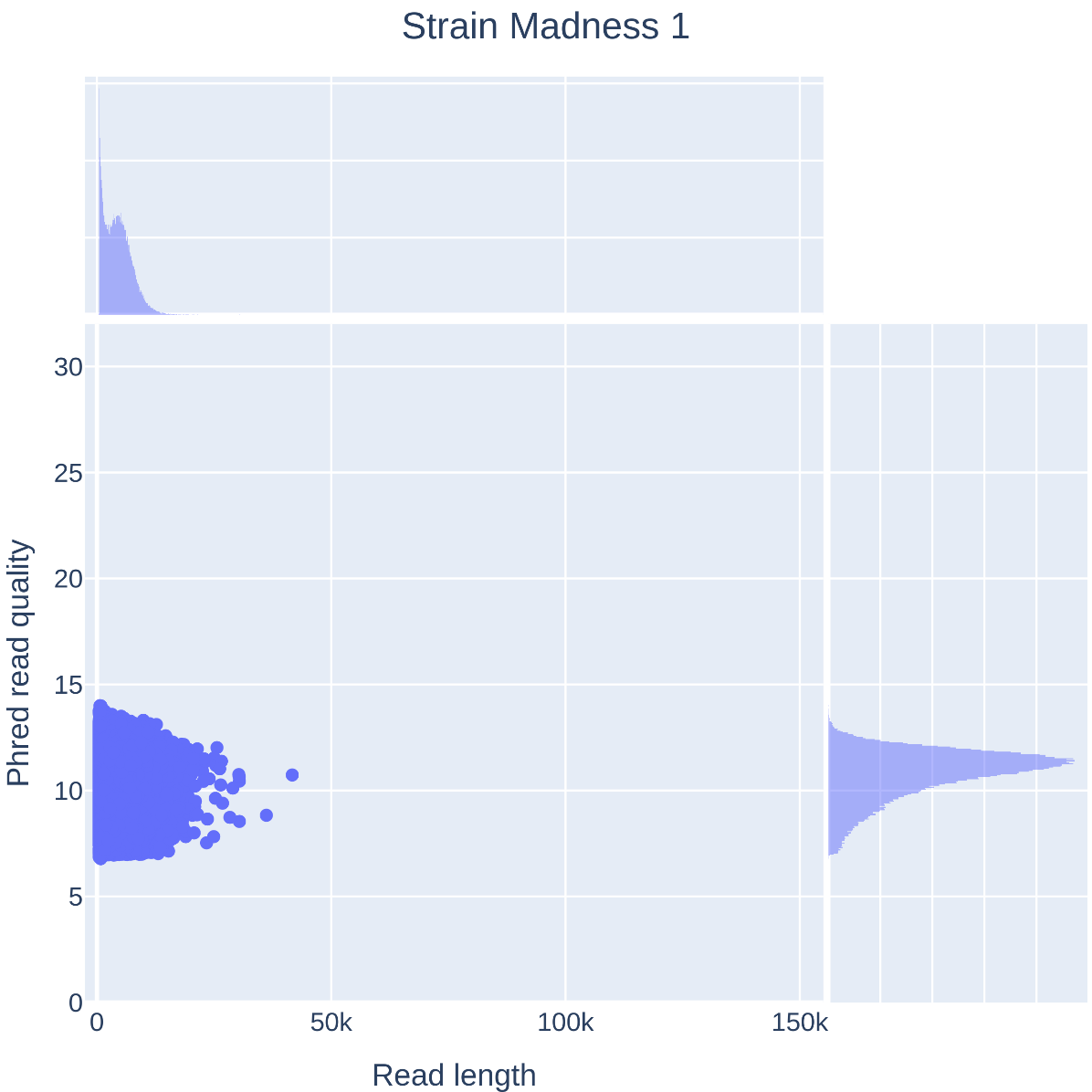


Figure S12: A scatter plot of the reads for Strain Madness 1. The x-axis represents the length while the y-axis represents the mean quality of each read. The histograms of the length and quality are shown at the top and right side, respectively. A random subsample of 100,000 reads is plotted.

## Figure S13


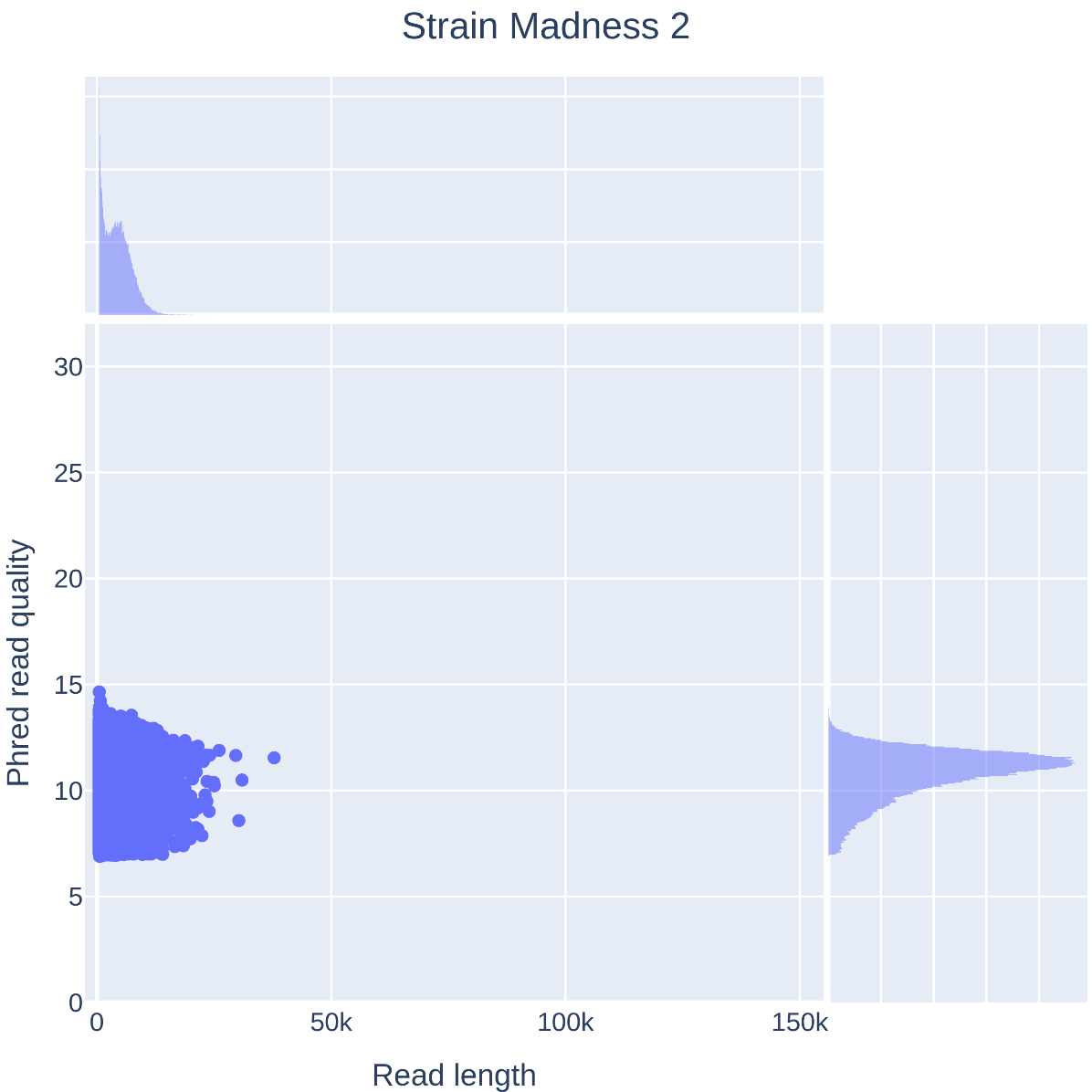


Figure S13: A scatter plot of the reads for Strain Madness 2. The x-axis represents the length while the y-axis represents the mean quality of each read. The histograms of the length and quality are shown at the top and right side, respectively. A random subsample of 100,000 reads is plotted.

## Figure S14


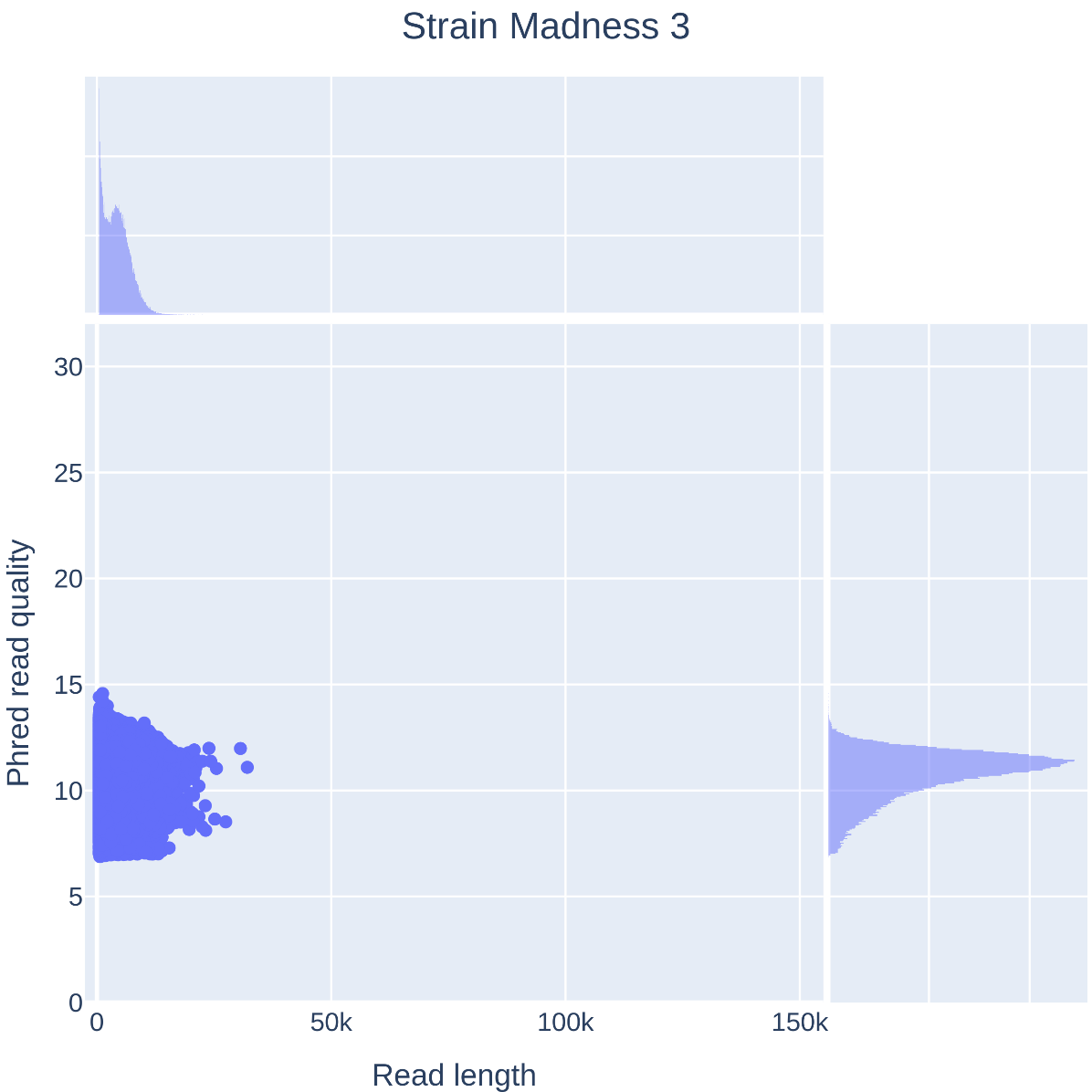


Figure S14: A scatter plot of the reads for Strain Madness 3. The x-axis represents the length while the y-axis represents the mean quality of each read. The histograms of the length and quality are shown at the top and right side, respectively. A random subsample of 100,000 reads is plotted.

## Figure S15


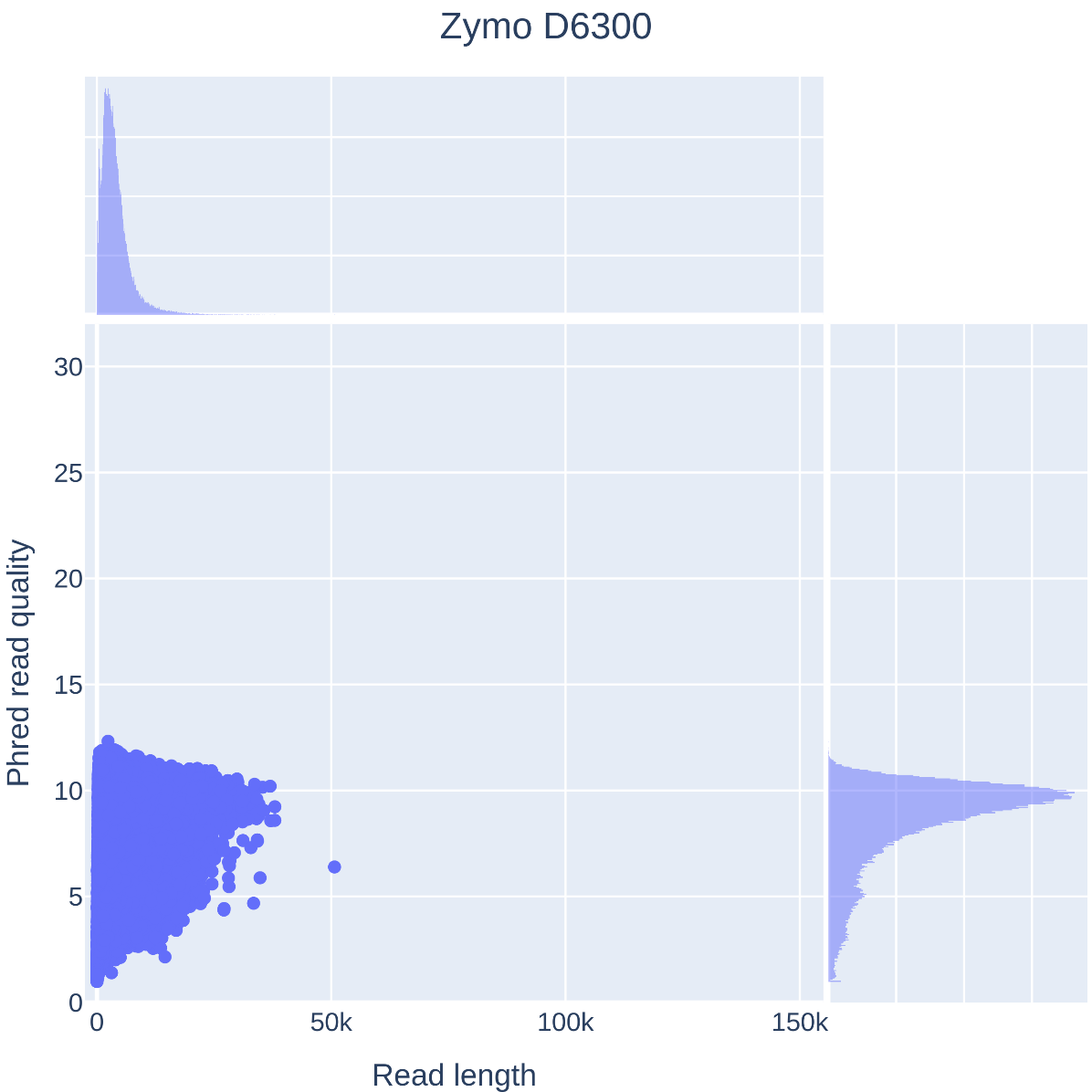


Figure S15: A scatter plot of the reads for Zymo D6300. The x-axis represents the length while the y-axis represents the mean quality of each read. The histograms of the length and quality are shown at the top and right side, respectively. A random subsample of 100,000 reads is plotted.

## Figure S16


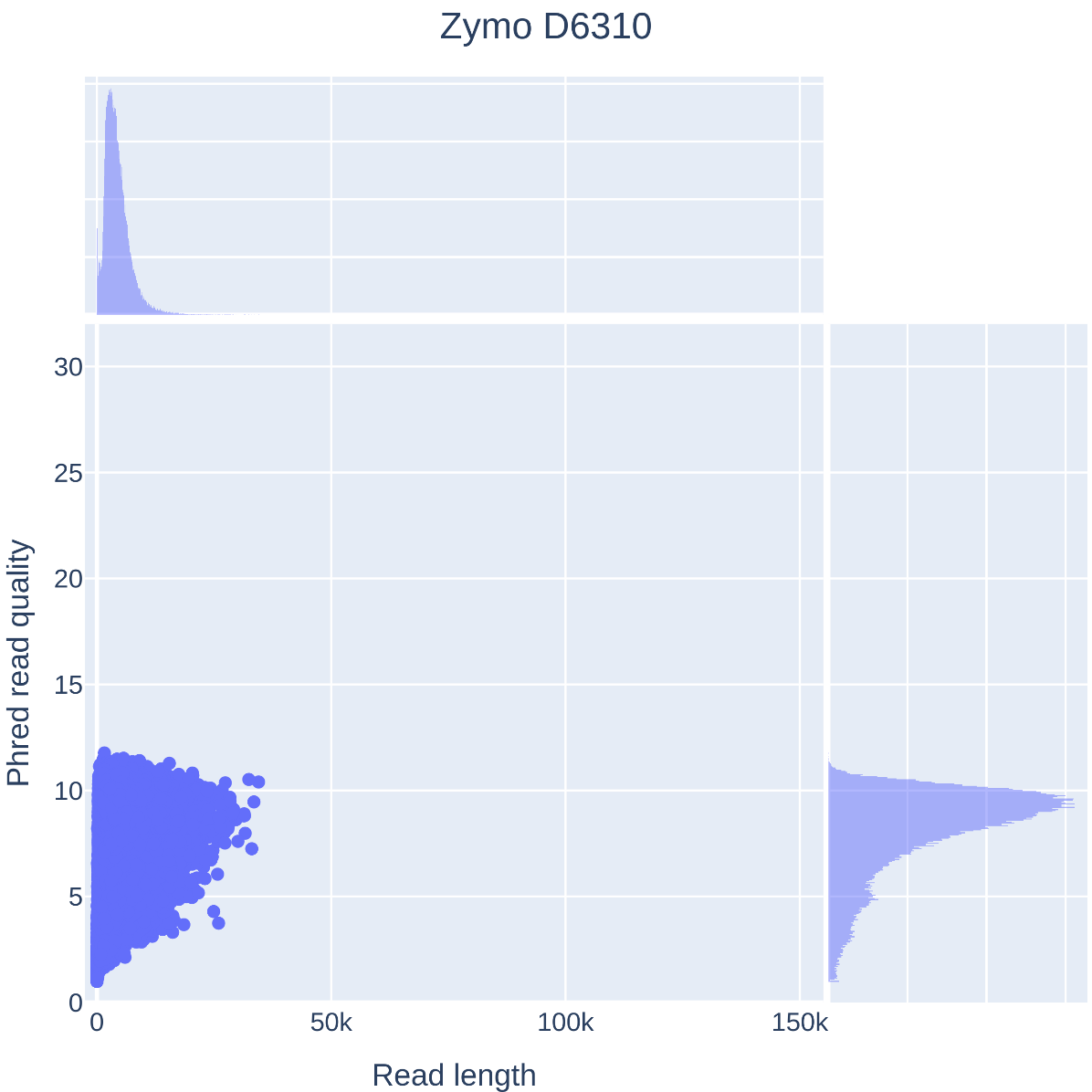


Figure S16: A scatter plot of the reads for Zymo D6310. The x-axis represents the length while the y-axis represents the mean quality of each read. The histograms of the length and quality are shown at the top and right side, respectively. A random subsample of 100,000 reads is plotted.

## Figure S17


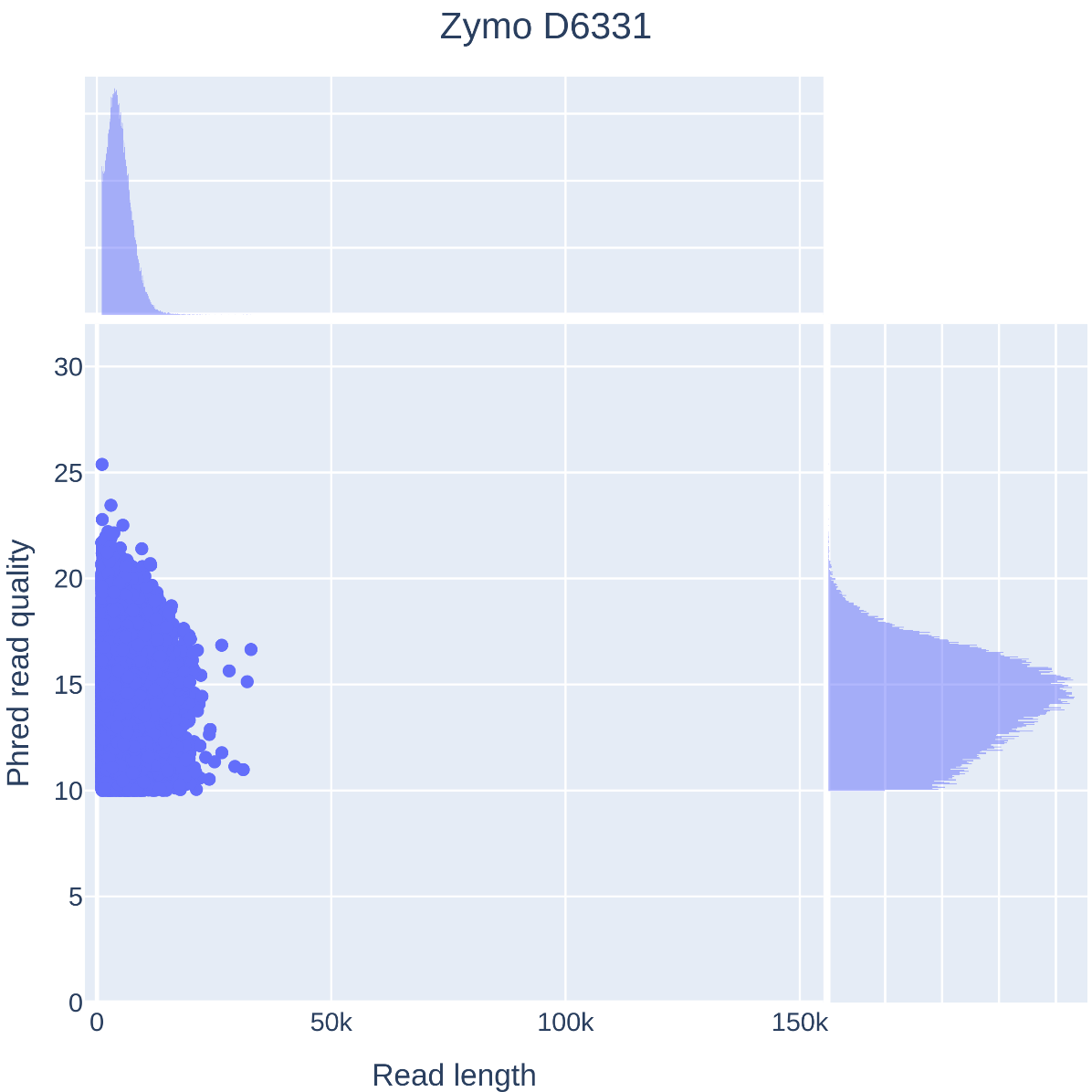


Figure S17: A scatter plot of the reads for Zymo D6331. The x-axis represents the length while the y-axis represents the mean quality of each read. The histograms of the length and quality are shown at the top and right side, respectively. A random subsample of 100,000 reads is plotted.

# Tables

## Table S1

| GENUS | DMC | Genomic | Protein | Marker mOTU | Marker Metaphlan |
| --- | --- | --- | --- | --- | --- |
|  | Zymo Research D6300 | 10/10 | 10/10 | 8/10 | 10/10 |
|  | Zymo Research D6310 | 10/10 | 10/10 | 8/10 | 10/10 |
|  | Zymo Research D6322 | 8/8 | 8/8 | 7/8 | 8/8 |
|  | Zymo Research D6331 | 17/17 | 17/17 | 15/17 | 17/17 |
|  | Bei Resources HM-276D | 17/17 | 17/17 | 17/17 | 17/17 |
|  | Bei Resources HM-277D | 17/17 | 17/17 | 17/17 | 17/17 |
|  | Sevim, V., Lee, J., Egan, R. et al. | 55/57 | 57/57 | 57/57 | 56/57 |
|  |  | 68/70 | 70/70 | 70/70 | 69/70 |
|  |  | 50/52 | 52/52 | 52/52 | 51/52 |

| SPECIES | DMC | Genomic | Protein | Marker mOTUs2 | Marker Metaphlan3 |
| --- | --- | --- | --- | --- | --- |
|  | Zymo Research D6300 | 10/10 | 10/10 | 8/10 | 8/10 |
|  | Zymo Research D6310 | 10/10 | 10/10 | 8/10 | 8/10 |
|  | Zymo Research D6322 | 8/8 | 8/8 | 7/8 | 6/8 |
|  | Zymo Research D6331 | 17/17 | 17/17 | 15/17 | 17/17 |
|  | Bei Resources HM-276D | 20/20 | 20/20 | 18/20 | 18/20 |
|  | Bei Resources HM-277D | 20/20 | 20/20 | 18/20 | 18/20 |
|  | Sevim, V., Lee, J., Egan, R. et al. | 63/69 | 69/69 | 62/69 | 62/69 |
|  |  | 79/85 | 84/85 | 76/85 | 76/85 |
|  |  | 55/62 | 60/62 | 55/62 | 56/62 |

**Table S1: The number of present genera and species in the databases for every DMC.** For each sample (row) and database (column), a fraction is displayed. The first table and second table show fractions for the genera and the species, respectively, in the samples and databases. The denominator displays the number of genera/species in the ground truth for the DMC. The nominator shows the number of genera/species from the ground truth that is also contained in the database. The fraction equals the maximum recall that can be achieved by the classifier types for a certain DMC.

## Table S2

| DMC | Kraken2 | KMA | Centrifuge | Kaiju | MMSeqs2 | Metaphlan3 | mOTUs2 |
| --- | --- | --- | --- | --- | --- | --- | --- |
| Zymo Research D6300 | 0-0 | 0-0 | 0-0 | 0-0 | 0-0 | 2-2 | 2-2 |
| Zymo Research D6310 | 0-0 | 0-0 | 0-0 | 0-0 | 1-0 | 9-2 | 7-2 |
| Zymo Research D6322 | 0-0 | 0-0 | 0-0 | 0-0 | 0-0 | 2-2 | 1-1 |
| Zymo Research D6331 | 1-0 | 1-0 | 1-0 | 1-0 | 2-0 | 4-0 | 6-2 |
| Bei Resources HM-276D | 0-0 | 0-0 | 0-0 | 0-0 | 0-0 | 3-2 | 4-2 |
| Bei Resources HM-277D | 0-0 | 0-0 | 0-0 | 0-0 | 0-0 | 9-2 | 8-2 |
| Strain Madness 1 | 7-6 | 8-6 | 6-6 | 3-0 | 7-0 | 24-7 | 33-7 |
| Strain Madness 2 | 7-6 | 8-6 | 6-6 | 4-1 | 14-1 | 40-9 | 48-9 |
| Strain Madness 3 | 5-5 | 7-5 | 5-5 | 2-0 | 8-0 | 22-6 | 29-7 |
| TOTAL | 3 | 7 | 1 | 9 | 31 | 83 | 104 |

**Table S2*:* Each cell represent for every DMC and classifier the subtraction of the number of FNs and the number of missing species in the database.** For every dataset (row) and classifier (column), the first number in the equation represent the number of FNs for a certain DMC and classifier. The number after the subtraction symbol represents the number of missing species in the database of a classifier for a certain DMC. Solving each equation represent the number of FNs of a classifier for a DMC should all missing species be present in the database and correctly classified by the classifier. The last column shows the total of all DMCs per classifier. Bracken and CCMetagen are omitted, they have the same values as Kraken2 and KMA, respectively.

## Table S3

|  | Starting material | Taxid | Super kingdom | Species | Relative abundance |
| --- | --- | --- | --- | --- | --- |
| Bei Resources HM-276D | DNA | 470 | Bacteria | Acinetobacter baumannii | 0.04815 |
|  |  | 1396 | Bacteria | Bacillus cereus | 0.03163 |
|  |  | 1063 | Bacteria | Cereibacter sphaeroides | 0.10004 |
|  |  | 1520 | Bacteria | Clostridium beijerinckii | 0.03114 |
|  |  | 1747 | Bacteria | Cutibacterium acnes | 0.062 |
|  |  | 1299 | Bacteria | Deinococcus radiodurans | 0.07412 |
|  |  | 1351 | Bacteria | Enterococcus faecalis | 0.04975 |
|  |  | 562 | Bacteria | Escherichia coli | 0.04817 |
|  |  | 210 | Bacteria | Helicobacter pylori | 0.06058 |
|  |  | 1596 | Bacteria | Lactobacillus gasseri | 0.02294 |
|  |  | 1639 | Bacteria | Listeria monocytogenes | 0.03565 |
|  |  | 487 | Bacteria | Neisseria meningitidis | 0.04127 |
|  |  | 821 | Bacteria | Phocaeicola vulgatus | 0.05358 |
|  |  | 287 | Bacteria | Pseudomonas aeruginosa | 0.11377 |
|  |  | 1660 | Bacteria | Schaalia odontolytica | 0.05796 |
|  |  | 1280 | Bacteria | Staphylococcus aureus | 0.04174 |
|  |  | 1282 | Bacteria | Staphylococcus epidermidis | 0.03631 |
|  |  | 1311 | Bacteria | Streptococcus agalactiae | 0.02242 |
|  |  | 1309 | Bacteria | Streptococcus mutans | 0.02954 |
|  |  | 1313 | Bacteria | Streptococcus pneumoniae | 0.03924 |

| Bei Resources HM-277D | DNA | 470 | Bacteria | Acinetobacter baumannii | NA |
| --- | --- | --- | --- | --- | --- |
|  |  | 1396 | Bacteria | Bacillus cereus | NA |
|  |  | 1063 | Bacteria | Cereibacter sphaeroides | NA |
|  |  | 1520 | Bacteria | Clostridium beijerinckii | NA |
|  |  | 1747 | Bacteria | Cutibacterium acnes | NA |
|  |  | 1299 | Bacteria | Deinococcus radiodurans | NA |
|  |  | 1351 | Bacteria | Enterococcus faecalis | NA |
|  |  | 562 | Bacteria | Escherichia coli | NA |
|  |  | 210 | Bacteria | Helicobacter pylori | NA |
|  |  | 1596 | Bacteria | Lactobacillus gasseri | NA |
|  |  | 1639 | Bacteria | Listeria monocytogenes | NA |
|  |  | 487 | Bacteria | Neisseria meningitidis | NA |
|  |  | 821 | Bacteria | Phocaeicola vulgatus | NA |
|  |  | 287 | Bacteria | Pseudomonas aeruginosa | NA |
|  |  | 1660 | Bacteria | Schaalia odontolytica | NA |
|  |  | 1280 | Bacteria | Staphylococcus aureus | NA |
|  |  | 1282 | Bacteria | Staphylococcus epidermidis | NA |
|  |  | 1311 | Bacteria | Streptococcus agalactiae | NA |
|  |  | 1309 | Bacteria | Streptococcus mutans | NA |
|  |  | 1313 | Bacteria | Streptococcus pneumoniae | NA |

| Strain Madness 1 | DNA and cells | 242703 | Archaea | Acidilobus saccharovorans | 0.0003 |
| --- | --- | --- | --- | --- | --- |
|  |  | 2234 | Archaea | Archaeoglobus fulgidus | 0.0117 |
|  |  | 379547 | Archaea | Candidatus Aciduliprofundum boonei | 0.0003 |
|  |  | 2242 | Archaea | Halobacterium salinarum | 0.0253 |
|  |  | 2246 | Archaea | Haloferax volcanii | 0.0005 |
|  |  | 160233 | Archaea | Ignicoccus hospitalis | 0.0284 |
|  |  | 54259 | Archaea | Ignicoccus islandicus | 0.011 |
|  |  | 66851 | Archaea | Methanobrevibacter oralis | 0.0076 |
|  |  | 2190 | Archaea | Methanocaldococcus jannaschii | 0.0565 |
|  |  | 39152 | Archaea | Methanococcus maripaludis | 0.0161 |
|  |  | 1080712 | Archaea | Methanomassiliicoccus luminyensis | 0.0006 |
|  |  | 101192 | Archaea | Methanomethylovorans hollandica | 0.0005 |
|  |  | 2320 | Archaea | Methanopyrus kandleri | 0.0021 |
|  |  | 2214 | Archaea | Methanosarcina acetivorans | 0.0018 |
|  |  | 2180 | Archaea | Methanothermus fervidus | 0.0718 |
|  |  | 160232 | Archaea | Nanoarchaeum equitans | 0.0096 |
|  |  | 13773 | Archaea | Pyrobaculum aerophilum | 0.0023 |
|  |  | 121277 | Archaea | Pyrobaculum arsenaticum | 0.0005 |
|  |  | 181486 | Archaea | Pyrobaculum calidifontis | 0.0254 |
|  |  | 2261 | Archaea | Pyrococcus furiosus | 0.004 |
|  |  | 53953 | Archaea | Pyrococcus horikoshii | 0.0586 |
|  |  | 111955 | Archaea | Sulfurisphaera tokodaii | 0.0008 |
|  |  | 1515 | Bacteria | Acetivibrio thermocellus | 0.0027 |
|  |  | 33075 | Bacteria | Acidobacterium capsulatum | 0.002 |
|  |  | 239935 | Bacteria | Akkermansia muciniphila | 0.0091 |
|  |  | 818 | Bacteria | Bacteroides thetaiotaomicron | 0.0032 |
|  |  | 520 | Bacteria | Bordetella pertussis | 0.0048 |
|  |  | 31899 | Bacteria | Caldicellulosiruptor bescii | 0.0021 |
|  |  | 44001 | Bacteria | Caldicellulosiruptor saccharolyticus | 0.0089 |
|  |  | 1097 | Bacteria | Chlorobaculum tepidum | 0.0024 |
|  |  | 1092 | Bacteria | Chlorobium limicola | 0.0232 |
|  |  | 1096 | Bacteria | Chlorobium phaeobacteroides | 0.0069 |
|  |  | 1094 | Bacteria | Chlorobium phaeovibrioides | 0.0129 |
|  |  | 1108 | Bacteria | Chloroflexus aurantiacus | 0.002 |
|  |  | 225194 | Bacteria | Citrifermentans bemidjiense | 0.000033 |
|  |  | 1299 | Bacteria | Deinococcus radiodurans | 0.0062 |
|  |  | 36854 | Bacteria | Desulfitobacterium dehalogenans | 0.000033 |
|  |  | 1986146 | Bacteria | Desulfobulbus oralis | 0.0735 |
|  |  | 901 | Bacteria | Desulfovibrio piger | 0.0439 |
|  |  | 881 | Bacteria | Desulfovibrio vulgaris | 0.0003 |
|  |  | 513050 | Bacteria | Dictyoglomus turgidum | 0.0069 |
|  |  | 1351 | Bacteria | Enterococcus faecalis | 0.0015 |
|  |  | 851 | Bacteria | Fusobacterium nucleatum | 0.0281 |
|  |  | 173480 | Bacteria | Gemmatimonas aurantiaca | 0.0241 |
|  |  | 35554 | Bacteria | Geobacter sulfurreducens | 0.0059 |
|  |  | 65 | Bacteria | Herpetosiphon aurantiacus | 0.0165 |
|  |  | 380749 | Bacteria | Hydrogenobaculum sp. Y04AAS1 | 0.0163 |
|  |  | 34029 | Bacteria | Leptothrix cholodnii | 0.0005 |
|  |  | 915 | Bacteria | Nitrosomonas europaea | 0.0568 |
|  |  | 103690 | Bacteria | Nostoc sp. PCC 7120 = FACHB-418 | 0.0137 |
|  |  | 36873 | Bacteria | Paraburkholderia xenovorans | 0.0058 |
|  |  | 309805 | Bacteria | Persephonella marina | 0.0281 |
|  |  | 821 | Bacteria | Phocaeicola vulgatus | 0.0047 |
|  |  | 837 | Bacteria | Porphyromonas gingivalis | 0.0217 |
|  |  | 641491 | Bacteria | Pseudodesulfovibrio mercurii | 0.0005 |
|  |  | 294 | Bacteria | Pseudomonas fluorescens | 0.0107 |
|  |  | 303 | Bacteria | Pseudomonas putida | 0.0148 |
|  |  | 265606 | Bacteria | Rhodopirellula baltica | 0.0132 |
|  |  | 89184 | Bacteria | Ruegeria pomeroyi | 0.0007 |
|  |  | 168697 | Bacteria | Salinispora arenicola | 0.0039 |
|  |  | 168695 | Bacteria | Salinispora tropica | 0.0049 |
|  |  | 62322 | Bacteria | Shewanella baltica | 0.039 |
|  |  | 359303 | Bacteria | Shewanella loihica | 0.0055 |
|  |  | 436114 | Bacteria | Sulfurihydrogenibium sp. YO3AOP1 | 0.0069 |
|  |  | 496866 | Bacteria | Thermoanaerobacter pseudethanolicus | 0.0248 |
|  |  | 2337 | Bacteria | Thermotoga neapolitana | 0.0161 |
|  |  | 93929 | Bacteria | Thermotoga petrophila | 0.0695 |
|  |  | 158 | Bacteria | Treponema denticola | 0.0127 |
|  |  | 844 | Bacteria | Wolinella succinogenes | 0.0066 |

| Strain Madness 2 | DNA and cells | 242703 | Archaea | Acidilobus saccharovorans | 0.0003 |
| --- | --- | --- | --- | --- | --- |
|  |  | 2234 | Archaea | Archaeoglobus fulgidus | 0.0115 |
|  |  | 379547 | Archaea | Candidatus Aciduliprofundum boonei | 0.0003 |
|  |  | 2242 | Archaea | Halobacterium salinarum | 0.025 |
|  |  | 2246 | Archaea | Haloferax volcanii | 0.0005 |
|  |  | 160233 | Archaea | Ignicoccus hospitalis | 0.028 |
|  |  | 54259 | Archaea | Ignicoccus islandicus | 0.0108 |
|  |  | 66851 | Archaea | Methanobrevibacter oralis | 0.0075 |
|  |  | 2190 | Archaea | Methanocaldococcus jannaschii | 0.0557 |
|  |  | 39152 | Archaea | Methanococcus maripaludis | 0.0159 |
|  |  | 1080712 | Archaea | Methanomassiliicoccus luminyensis | 0.0006 |
|  |  | 101192 | Archaea | Methanomethylovorans hollandica | 0.0005 |
|  |  | 2320 | Archaea | Methanopyrus kandleri | 0.0021 |
|  |  | 2214 | Archaea | Methanosarcina acetivorans | 0.0017 |
|  |  | 2180 | Archaea | Methanothermus fervidus | 0.0708 |
|  |  | 160232 | Archaea | Nanoarchaeum equitans | 0.0094 |
|  |  | 13773 | Archaea | Pyrobaculum aerophilum | 0.0023 |
|  |  | 121277 | Archaea | Pyrobaculum arsenaticum | 0.0005 |
|  |  | 181486 | Archaea | Pyrobaculum calidifontis | 0.0251 |
|  |  | 2261 | Archaea | Pyrococcus furiosus | 0.004 |
|  |  | 53953 | Archaea | Pyrococcus horikoshii | 0.0577 |
|  |  | 111955 | Archaea | Sulfurisphaera tokodaii | 0.0007 |
|  |  | 1515 | Bacteria | Acetivibrio thermocellus | 0.0027 |
|  |  | 33075 | Bacteria | Acidobacterium capsulatum | 0.002 |
|  |  | 470 | Bacteria | Acinetobacter baumannii | 0.0005 |
|  |  | 239935 | Bacteria | Akkermansia muciniphila | 0.0089 |
|  |  | 1396 | Bacteria | Bacillus cereus | 0.0004 |
|  |  | 818 | Bacteria | Bacteroides thetaiotaomicron | 0.0032 |
|  |  | 1680 | Bacteria | Bifidobacterium adolescentis | 0.001 |
|  |  | 520 | Bacteria | Bordetella pertussis | 0.0047 |
|  |  | 31899 | Bacteria | Caldicellulosiruptor bescii | 0.0021 |
|  |  | 44001 | Bacteria | Caldicellulosiruptor saccharolyticus | 0.0088 |
|  |  | 1063 | Bacteria | Cereibacter sphaeroides | 0.0004 |
|  |  | 1097 | Bacteria | Chlorobaculum tepidum | 0.0024 |
|  |  | 1092 | Bacteria | Chlorobium limicola | 0.0229 |
|  |  | 1096 | Bacteria | Chlorobium phaeobacteroides | 0.0068 |
|  |  | 1094 | Bacteria | Chlorobium phaeovibrioides | 0.0128 |
|  |  | 225194 | Bacteria | Citrifermentans bemidjiense | 0.000033 |
|  |  | 1108 | Bacteria | Chloroflexus aurantiacus | 0.002 |
|  |  | 1520 | Bacteria | Clostridium beijerinckii | 0.0003 |
|  |  | 1747 | Bacteria | Cutibacterium acnes | 0.0008 |
|  |  | 1299 | Bacteria | Deinococcus radiodurans | 0.0067 |
|  |  | 36854 | Bacteria | Desulfitobacterium dehalogenans | 0.000033 |
|  |  | 1986146 | Bacteria | Desulfobulbus oralis | 0.0725 |
|  |  | 901 | Bacteria | Desulfovibrio piger | 0.0433 |
|  |  | 881 | Bacteria | Desulfovibrio vulgaris | 0.0003 |
|  |  | 513050 | Bacteria | Dictyoglomus turgidum | 0.0068 |
|  |  | 1351 | Bacteria | Enterococcus faecalis | 0.0021 |
|  |  | 562 | Bacteria | Escherichia coli | 0.0004 |
|  |  | 851 | Bacteria | Fusobacterium nucleatum | 0.0277 |
|  |  | 173480 | Bacteria | Gemmatimonas aurantiaca | 0.0238 |
|  |  | 35554 | Bacteria | Geobacter sulfurreducens | 0.0058 |
|  |  | 210 | Bacteria | Helicobacter pylori | 0.0012 |
|  |  | 65 | Bacteria | Herpetosiphon aurantiacus | 0.0163 |
|  |  | 380749 | Bacteria | Hydrogenobaculum sp. Y04AAS1 | 0.0161 |
|  |  | 1596 | Bacteria | Lactobacillus gasseri | 0.0011 |
|  |  | 34029 | Bacteria | Leptothrix cholodnii | 0.0005 |
|  |  | 487 | Bacteria | Neisseria meningitidis | 0.0009 |
|  |  | 915 | Bacteria | Nitrosomonas europaea | 0.056 |
|  |  | 103690 | Bacteria | Nostoc sp. PCC 7120 = FACHB-418 | 0.0135 |
|  |  | 36873 | Bacteria | Paraburkholderia xenovorans | 0.0057 |
|  |  | 309805 | Bacteria | Persephonella marina | 0.0277 |
|  |  | 821 | Bacteria | Phocaeicola vulgatus | 0.005 |
|  |  | 837 | Bacteria | Porphyromonas gingivalis | 0.0223 |
|  |  | 641491 | Bacteria | Pseudodesulfovibrio mercurii | 0.0005 |
|  |  | 294 | Bacteria | Pseudomonas fluorescens | 0.0105 |
|  |  | 2994495 | Bacteria | Pseudomonas paraeruginosa | 0.0003 |
|  |  | 303 | Bacteria | Pseudomonas putida | 0.0146 |
|  |  | 265606 | Bacteria | Rhodopirellula baltica | 0.013 |
|  |  | 89184 | Bacteria | Ruegeria pomeroyi | 0.0007 |
|  |  | 168697 | Bacteria | Salinispora arenicola | 0.0039 |
|  |  | 168695 | Bacteria | Salinispora tropica | 0.0048 |
|  |  | 1660 | Bacteria | Schaalia odontolytica | 0.0008 |
|  |  | 62322 | Bacteria | Shewanella baltica | 0.0384 |
|  |  | 359303 | Bacteria | Shewanella loihica | 0.0055 |
|  |  | 1280 | Bacteria | Staphylococcus aureus | 0.0007 |
|  |  | 1282 | Bacteria | Staphylococcus epidermidis | 0.0008 |
|  |  | 1311 | Bacteria | Streptococcus agalactiae | 0.0009 |
|  |  | 1309 | Bacteria | Streptococcus mutans | 0.001 |
|  |  | 436114 | Bacteria | Sulfurihydrogenibium sp. YO3AOP1 | 0.0068 |
|  |  | 496866 | Bacteria | Thermoanaerobacter pseudethanolicus | 0.0245 |
|  |  | 2337 | Bacteria | Thermotoga neapolitana | 0.0158 |
|  |  | 93929 | Bacteria | Thermotoga petrophila | 0.0685 |
|  |  | 158 | Bacteria | Treponema denticola | 0.0125 |
|  |  | 844 | Bacteria | Wolinella succinogenes | 0.0065 |

| Strain Madness 3 | DNA and cells | 242703 | Archaea | Acidilobus saccharovorans | 0.0003 |
| --- | --- | --- | --- | --- | --- |
|  |  | 2234 | Archaea | Archaeoglobus fulgidus | 0.0092 |
|  |  | 2242 | Archaea | Halobacterium salinarum | 0.0249 |
|  |  | 2246 | Archaea | Haloferax volcanii | 0.0005 |
|  |  | 160233 | Archaea | Ignicoccus hospitalis | 0.0419 |
|  |  | 54259 | Archaea | Ignicoccus islandicus | 0.027 |
|  |  | 2190 | Archaea | Methanocaldococcus jannaschii | 0.1112 |
|  |  | 39152 | Archaea | Methanococcus maripaludis | 0.0144 |
|  |  | 2320 | Archaea | Methanopyrus kandleri | 0.0026 |
|  |  | 2214 | Archaea | Methanosarcina acetivorans | 0.0017 |
|  |  | 2180 | Archaea | Methanothermus fervidus | 0.0566 |
|  |  | 160232 | Archaea | Nanoarchaeum equitans | 0.0062 |
|  |  | 13773 | Archaea | Pyrobaculum aerophilum | 0.0011 |
|  |  | 181486 | Archaea | Pyrobaculum calidifontis | 0.005 |
|  |  | 53953 | Archaea | Pyrococcus horikoshii | 0.0721 |
|  |  | 111955 | Archaea | Sulfurisphaera tokodaii | 0.0007 |
|  |  | 1515 | Bacteria | Acetivibrio thermocellus | 0.0016 |
|  |  | 33075 | Bacteria | Acidobacterium capsulatum | 0.0012 |
|  |  | 239935 | Bacteria | Akkermansia muciniphila | 0.0125 |
|  |  | 818 | Bacteria | Bacteroides thetaiotaomicron | 0.002 |
|  |  | 520 | Bacteria | Bordetella pertussis | 0.0047 |
|  |  | 31899 | Bacteria | Caldicellulosiruptor bescii | 0.0021 |
|  |  | 44001 | Bacteria | Caldicellulosiruptor saccharolyticus | 0.0132 |
|  |  | 1097 | Bacteria | Chlorobaculum tepidum | 0.006 |
|  |  | 1092 | Bacteria | Chlorobium limicola | 0.0137 |
|  |  | 1096 | Bacteria | Chlorobium phaeobacteroides | 0.0068 |
|  |  | 1094 | Bacteria | Chlorobium phaeovibrioides | 0.0191 |
|  |  | 1108 | Bacteria | Chloroflexus aurantiacus | 0.001 |
|  |  | 1299 | Bacteria | Deinococcus radiodurans | 0.0046 |
|  |  | 1986146 | Bacteria | Desulfobulbus oralis | 0.0362 |
|  |  | 899 | Bacteria | Desulfomicrobium baculatum | 0.0004 |
|  |  | 901 | Bacteria | Desulfovibrio piger | 0.0648 |
|  |  | 881 | Bacteria | Desulfovibrio vulgaris | 0.0005 |
|  |  | 513050 | Bacteria | Dictyoglomus turgidum | 0.0054 |
|  |  | 1351 | Bacteria | Enterococcus faecalis | 0.003 |
|  |  | 851 | Bacteria | Fusobacterium nucleatum | 0.0138 |
|  |  | 173480 | Bacteria | Gemmatimonas aurantiaca | 0.0238 |
|  |  | 35554 | Bacteria | Geobacter sulfurreducens | 0.001 |
|  |  | 65 | Bacteria | Herpetosiphon aurantiacus | 0.026 |
|  |  | 380749 | Bacteria | Hydrogenobaculum sp. Y04AAS1 | 0.0129 |
|  |  | 915 | Bacteria | Nitrosomonas europaea | 0.028 |
|  |  | 103690 | Bacteria | Nostoc sp. PCC 7120 = FACHB-418 | 0.0203 |
|  |  | 36873 | Bacteria | Paraburkholderia xenovorans | 0.0028 |
|  |  | 34090 | Bacteria | Pelodictyon phaeoclathratiforme | 0.001 |
|  |  | 309805 | Bacteria | Persephonella marina | 0.0152 |
|  |  | 821 | Bacteria | Phocaeicola vulgatus | 0.0139 |
|  |  | 837 | Bacteria | Porphyromonas gingivalis | 0.0427 |
|  |  | 641491 | Bacteria | Pseudodesulfovibrio mercurii | 0.0003 |
|  |  | 294 | Bacteria | Pseudomonas fluorescens | 0.0035 |
|  |  | 303 | Bacteria | Pseudomonas putida | 0.0118 |
|  |  | 265606 | Bacteria | Rhodopirellula baltica | 0.0078 |
|  |  | 168697 | Bacteria | Salinispora arenicola | 0.0031 |
|  |  | 168695 | Bacteria | Salinispora tropica | 0.0039 |
|  |  | 62322 | Bacteria | Shewanella baltica | 0.0478 |
|  |  | 359303 | Bacteria | Shewanella loihica | 0.0076 |
|  |  | 436114 | Bacteria | Sulfurihydrogenibium sp. YO3AOP1 | 0.0055 |
|  |  | 496866 | Bacteria | Thermoanaerobacter pseudethanolicus | 0.0183 |
|  |  | 2336 | Bacteria | Thermotoga maritima | 0.0614 |
|  |  | 2337 | Bacteria | Thermotoga neapolitana | 0.0317 |
|  |  | 158 | Bacteria | Treponema denticola | 0.0156 |
|  |  | 844 | Bacteria | Wolinella succinogenes | 0.0054 |
|  |  | 542 | Bacteria | Zymomonas mobilis | 0.0005 |

| Zymo D6300 | Cells | 1423 | Bacteria | Bacillus subtilis | 0.12 |
| --- | --- | --- | --- | --- | --- |
|  |  | 1351 | Bacteria | Enterococcus faecalis | 0.12 |
|  |  | 562 | Bacteria | Escherichia coli | 0.12 |
|  |  | 1613 | Bacteria | Limosilactobacillus fermentum | 0.12 |
|  |  | 1639 | Bacteria | Listeria monocytogenes | 0.12 |
|  |  | 287 | Bacteria | Pseudomonas aeruginosa | 0.12 |
|  |  | 28901 | Bacteria | Salmonella enterica | 0.12 |
|  |  | 1280 | Bacteria | Staphylococcus aureus | 0.12 |
|  |  | 5207 | Eukaryota | Cryptococcus neoformans | 0.02 |
|  |  | 4932 | Eukaryota | Saccharomyces cerevisiae | 0.02 |

| Zymo D6310 | Cells | 1423 | Bacteria | Bacillus subtilis | 0.0089 |
| --- | --- | --- | --- | --- | --- |
|  |  | 1351 | Bacteria | Enterococcus faecalis | 0.0000089 |
|  |  | 562 | Bacteria | Escherichia coli | 0.00089 |
|  |  | 1613 | Bacteria | Limosilactobacillus fermentum | 0.000089 |
|  |  | 1639 | Bacteria | Listeria monocytogenes | 0.891 |
|  |  | 287 | Bacteria | Pseudomonas aeruginosa | 0.08900000 |
|  |  | 28901 | Bacteria | Salmonella enterica | 0.00089 |
|  |  | 1280 | Bacteria | Staphylococcus aureus | 0.00000089 |
|  |  | 5207 | Eukaryota | Cryptococcus neoformans | 0.0000089 |
|  |  | 4932 | Eukaryota | Saccharomyces cerevisiae | 0.0089 |

| Zymo D6322 | DNA | 1423 | Bacteria | Bacillus subtilis | 0.14 |
| --- | --- | --- | --- | --- | --- |
|  |  | 1351 | Bacteria | Enterococcus faecalis | 0.14 |
|  |  | 562 | Bacteria | Escherichia coli | 0.14 |
|  |  | 1639 | Bacteria | Listeria monocytogenes | 0.14 |
|  |  | 287 | Bacteria | Pseudomonas aeruginosa | 0.14 |
|  |  | 28901 | Bacteria | Salmonella enterica | 0.14 |
|  |  | 1280 | Bacteria | Staphylococcus aureus | 0.14 |
|  |  | 4932 | Eukaryota | Saccharomyces cerevisiae | 0.02 |

| Zymo D6331 | Cells | 2173 | Archaea | Methanobrevibacter smithii | 0.001 |
| --- | --- | --- | --- | --- | --- |
|  |  | 239935 | Bacteria | Akkermansia muciniphila | 0.015 |
|  |  | 817 | Bacteria | Bacteroides fragilis | 0.14 |
|  |  | 1680 | Bacteria | Bifidobacterium adolescentis | 0.06 |
|  |  | 1496 | Bacteria | Clostridioides difficile | 0.015 |
|  |  | 1502 | Bacteria | Clostridium perfringens | 0.000001 |
|  |  | 1351 | Bacteria | Enterococcus faecalis | 0.00001 |
|  |  | 562 | Bacteria | Escherichia coli | 0.14 |
|  |  | 853 | Bacteria | Faecalibacterium prausnitzii | 0.14 |
|  |  | 851 | Bacteria | Fusobacterium nucleatum | 0.06 |
|  |  | 1613 | Bacteria | Limosilactobacillus fermentum | 0.06 |
|  |  | 28128 | Bacteria | Prevotella corporis | 0.06 |
|  |  | 301301 | Bacteria | Roseburia hominis | 0.14 |
|  |  | 28901 | Bacteria | Salmonella enterica | 0.0001 |
|  |  | 423477 | Bacteria | Veillonella rogosae | 0.14 |
|  |  | 5476 | Eukaryota | Candida albicans | 0.015 |
|  |  | 4932 | Eukaryota | Saccharomyces cerevisiae | 0.014 |

**Table S3: Composition of each DMC. The first column lists the name of each DMC.** The second column states whether the DMC was originally available as cells or DNA. The third column displays the taxonomic ID of the present organism. The fourth and fifth column shows the superkingdom and species. The last column shows the relative abundance of each organism.

# References

1. Altschul, S. F., Gish, W., Miller, W., Myers, E. W. & Lipman, D. J. Basic local alignment search tool. *J. Mol. Biol.* **215**, 403–410 (1990).

2. Schoch, C. L. *et al.* NCBI Taxonomy: a comprehensive update on curation, resources and tools. *Database* **2020**, baaa062 (2020).

3. Shen, W. & Ren, H. TaxonKit: A practical and efficient NCBI taxonomy toolkit. *J. Genet. Genomics* **48**, 844–850 (2021).
